# Supplementary material for: SpiDe-Sr: blind super-resolution network for precise cell segmentation and clustering in spatial proteomics imaging
Source: Nat Commun. 2024 Mar 28;15:2708. doi: 10.1038/s41467-024-46989-z (PMC10978886; doi:10.1038/s41467-024-46989-z)
Supplement: Supplementary file 1 — Supplementary Information [file 41467_2024_46989_MOESM1_ESM.pdf]

Supplementary Information for

SpiDe-Sr: blind super-resolution network for precise cell segmentation and clustering in spatial proteomics imaging

Rui Chen[1, 2†], Jiasu Xu[1, 2†], Boqian Wang[1, 2], Yi Ding[1, 2], Aynur Abdulla[1], Yiyang Li[2], Lai Jiang[1], Xianting Ding[1, 2*]

[1] Department of Anesthesiology and Surgical Intensive Care Unit, Xinhua Hospital, School of Medicine and School of Biomedical Engineering, Shanghai Jiao Tong University, Shanghai, China

[2] State Key Laboratory of Systems Medicine for Cancer, Institute for Personalized Medicine, Shanghai Jiao Tong University, Shanghai, China

[†] These authors contributed equally.

[*] Corresponding author, e-mail: dingxianting@sjtu.edu.cn.

This PDF file includes:

Glossary of Abbreviations

Supplementary Notes 1-7

Supplementary Tables 1-10

Supplementary Figures 1-12

Supplementary References

Contents

Section **Page**

Supplementary Notes 5

Supplementary Note 1. Theorem Proof 5

Supplementary Note 2. Pseudo Code 7

Supplementary Note 3. Sample Pre-staining for Laser Microdissection 8

Supplementary Note 4. Laser Microdissection of Bacterial Enrichment Regions 9

Supplementary Note 5. Label-free Quantitative Proteomics 10

Supplementary Note 6. Analysis of label-free proteomics data 12

Supplementary Note 7. Calculate the accuracy of cell extraction 13

Supplementary Tables 14

Supplementary Table 1. Antibody Information 14

Supplementary Table 2. Reagent Information 15

Supplementary Table 3. Program Operating Environment 16

Supplementary Table 4. Significantly different proteins (Top 25) in bacterial enrichment regions of HER2 breast cancer samples 17

Supplementary Table 5. Significantly different proteins (Top 25) in bacterial enrichment regions of LA breast cancer samples 18

Supplementary Table 6. Significantly different proteins (Top 25) in bacterial enrichment regions of LB breast cancer samples 19

Supplementary Table 7. Significantly different proteins (Top 25) in bacterial enrichment regions of TNBC breast cancer samples 20

Supplementary Table 8. Correlation between LPS expression and other biomarker expressions 21

Supplementary Table 9. Correlation between LTA expression and other biomarker expressions 22

Supplementary Table 10. Default parameters for Cellpose 23

Supplementary Figures 24

Supplementary Fig. 1 The architecture of the U_θ and training process of denoising module. 25

Supplementary Fig. 2 The architecture of the super-resolution (SR) module. 27

Supplementary Fig. 3 The sensitivity of super-resolution to blur kernel mismatch and test results for different noisy scenarios. 29

Supplementary Fig. 4 Validation of different SR methods (SRCNN/ KernelGAN/ RCAN/ SpiDe-Sr) on IMC images of cells. 31

Supplementary Fig. 5 Validation of different SR methods (SRCNN/ KernelGAN/ RCAN/ SpiDe-Sr) on IMC images of mouse fatty liver tissues. 33

Supplementary Fig. 6 Validation of different SR methods (SRCNN/ KernelGAN/ RCAN/ SpiDe-Sr) on IMC images of human breast cancer tissues. 35

Supplementary Fig. 7 Application of SpiDe-Sr to spatial proteomics data of four major subtypes of breast cancer patients. 37

Supplementary Fig. 8 Analysis of raw spatial proteomics data (without SpiDe-Sr enhancement) from patients with four major subtypes of breast tumor. 39

Supplementary Fig. 9 Acquisition and analysis of label-free proteomics with clinical breast cancer specimens. 41

Supplementary Fig. 10 Functional protein signaling pathways and protein-protein interaction network from label-free proteomics data. 43

Supplementary Fig. 11 Four features output from each of the 9 middle layers of denoising module. 44

Supplementary Fig. 12 Western blot information. 45

Supplementary References 466

Glossary of Abbreviations

Technical Abbreviations

IMC: Imaging Mass Cytometry

PSNR: Peak Signal-to-Noise Ratio

SSIM: Structural Similarity

SR: Super-Resolution

LR: Low-Resolution

HR: High-Resolution

GT: Ground Truth

SRCNN: Super-Resolution Convolutional Neural Network

KernelGAN: Kernel Generative Adversarial Network

RCAN: Residual Channel Attention Network

SOTA: State-Of-The-Art.

OE: Operating Environment

SFTMD: Spatial Feature Transformer Multiple Degradations

IoU: Intersection over Union

NA: Numerical Aperture

CH: Calinski-Harabasz score

DB: Davies-Bouldin score

t-SNE: *t*-distributed Stochastic Neighbor Embedding

Conv: Convolution computing layer

PPI: Protein-Protein Interaction

Biological Abbreviations

PFA: Paraformaldehyde

IAA: Iodoacetamide

FA: Formic Acid

SDS: Sodium Dodecylsulfate

DTT: DL-Dithiothreitol

LC-MS/ MS: Liquid Chromatography-tandem Mass Spectrometry

G^-: Gram-Negative Bacteria

G^+: Gram-Positive Bacteria

HER2: Human Epidermal Growth Factor Receptor 2

LA: Luminal A

LB: Luminal B

TNBC: Triple Negative Breast Cancer

FFPE: Formalin Fixed Paraffin Embedded

LPS: Lipopolysaccharide

LTA: Lipteihcoicacid

ER: Estrogen Receptor

IFI6: Interferon alpha-inducible protein 6

ISG15: Interferon-Stimulating Gene 15

Ki67: marker of proliferation Ki-67

PKCD: Prkcd - protein kinase C, delta Gene

PR: Pathogenesis-Related Protein

GO: Gene Ontology

KEGG: Kyoto Encyclopedia of Genes and Genomes

Other Abbreviations

RT: Room Temperature

Supplementary Notes

Supplementary Note 1. Theorem Proof

There are two images y and z with the same ground truth x. The gap between the underlying clean images is

$$\varepsilon = \mathrm{E}_{z|x}(z) - \mathrm{E}_{y|x}(y) \neq 0.$$

Theorem Let y and z be two independent noisy images conditioned on x, and assume that there exists an $\varepsilon \neq 0$ such that $\mathrm{E}_{y|x}(y) = x$ and $\mathrm{E}_{z|x}(z) = x + \varepsilon$. Let the variance of z be σ_z^2. Then the following equation holds:

$$\mathrm{E}_{x,y}\|f_\theta(y) - x\|_2^2 = \mathrm{E}_{x,y,z}\|f_\theta(y) - z\|_2^2 - \sigma_z^2 + 2\,\varepsilon\,\mathrm{E}_{x,y}(f_\theta(y) - x).$$

Proof

$$\mathrm{E}_{x,y}\|f_\theta(y) - x\|_2^2 = \mathrm{E}_{y,z|x}\|f_\theta(y) - z + z - x\|_2^2$$

$$= \mathrm{E}_{y,z|x}\|f_\theta(y) - z\|_2^2 + \mathrm{E}_{z|x}\|z - x\|_2^2 + 2\,\mathrm{E}_{y,z|x}\|f_\theta(y) - z\|^{\mathrm{T}}(z - x)$$

$$= \mathrm{E}_{y,z|x}\|f_\theta(y) - z\|_2^2 + \sigma_z^2 + 2\,\mathrm{E}_{y,z|x}(f_\theta(y) - x + x - z)^{\mathrm{T}}(z - x)$$

$$= \mathrm{E}_{y,z|x}\|f_\theta(y) - z\|_2^2 + \sigma_z^2 + 2\,\mathrm{E}_{y,z|x}(f_\theta(y) - x)^{\mathrm{T}}(z - x) + 2\,\mathrm{E}_{z|x}(x - z)^{\mathrm{T}}(z - x)$$

$$= \mathrm{E}_{y,z|x}\|f_\theta(y) - z\|_2^2 - \sigma_z^2 + 2\,\mathrm{E}_{y,z|x}(f_\theta(y) - x)^{\mathrm{T}}(z - x)$$

Since y and z given x are independent of each other, the following equation holds:

$$\mathrm{E}_{y|x}\|f_\theta(y) - x\|_2^2 = \mathrm{E}_{y,z|x}\|f_\theta(y) - z\|_2^2 - \sigma_z^2 + 2\,\mathrm{E}_{y|x}(f_\theta(y) - x)^{\mathrm{T}}\,\mathrm{E}_{z|x}(z - x)$$

$$= \mathrm{E}_{y,z|x}\|f_\theta(y) - z\|_2^2 - \sigma_z^2 + 2\,\varepsilon\,\mathrm{E}_{y|x}(f_\theta(y) - x)$$

Since $\mathrm{E}_{x,y} = \mathrm{E}_x\,\mathrm{E}_{y|x}$, the following equation holds:

$$\mathrm{E}_{x,y}\|f_\theta(y) - x\|_2^2 = \mathrm{E}_{x,y,z}\|f_\theta(y) - z\|_2^2 - \sigma_z^2 + 2\,\varepsilon\,\mathrm{E}_{x,y}(f_\theta(y) - x).$$

Theorem states that when the gap $\varepsilon \neq 0$, since $\mathrm{E}_{x,y}(f_\theta(y) - x) \neq 0$, optimizing $\mathrm{E}_{x,y,z}\|f_\theta(y) - z\|_2^2$ does not yield the same solution as the supervised training loss

$E_{x,y}\|f_\theta(y) - x\|_2^2$. If $\varepsilon \to 0$, which means the gap is sufficiently small, $2\,\varepsilon\, E_{x,y}(f_\theta(y) - x) \to 0$, so the network trained with paired noisy image y and z works as a reasonable approximate solution to the supervised training network. For detailed discussions, refer to references[6, 7, 8].

Noise with spatial correlation is not taken into primary account in the network construction since existing studies generally assume that the noise is independently sampled[9,10]. And for IMC images, the distribution of proteins is generally correlated with the spatial structure of the specimen. If the noise has spatial correlations, the denoising module may encounter issues in distinguishing whether the single is a valid protein expression signal or simply just noise. Fortunately, our experimental results show that SpiDe-Sr is practically suitable for IMC images.

Supplementary Note 2. Pseudo Code

Step		SpiDe-Sr
	**Input:**	A set of raw noisy images $Y = \{y_i\}_{i=1}^{n}$;
		Denoising network U_θ;
		Hyper-parameter γ;
		Neighbor sub-sampler $G = (g_1, g_2)$;
		Predictor P_θ ;
		Corrector C_θ;
		SR network S_θ;
	**While**	not converged **do**
1		Input a raw noisy image $y \in Y$;
2		Generate sub-sampled noisy image pairs $(\mathrm{g}_1(\mathrm{y}), \mathrm{g}_2(\mathrm{y}))$ by G, $\mathrm{g}_1(\mathrm{y})$ is the network input and $\mathrm{g}_2(\mathrm{y})$ is the network target;
3		Input $g_1(y)$ into the U_θ to obtain the denoised image $U_\theta(g_1(y))$;
4		Calculate L_{rec}= $\|U_\theta(g_1(y) - g_2(y))\|_2^2$;
5		Input raw noisy image y into the U_θ to obtain the denoised image without gradients $U_\theta(y)$;
6		Generate sub-sampled denoised image pairs $(\mathrm{g}_1(U_\theta(y)), \mathrm{g}_2(U_\theta(y)))$;
7		Calculate $L_{reg} = \|U_\theta(g_1(y)) - g_2(y) - (g_1(U_\theta(y)) - g_2(U_\theta(y)))\|_2^2$;
8		Update denoising network U_θ by $L = L_{rec} + L_{reg}$;
	**end**	
9		Output denoised LR image I^{LR};
10		Initialize counter $i = 0$;
11		Predict the initial blur kernel $k_0 = P_\theta\ (I^{LR})$
12		Input k_0 and I^{LR} into S_θ to obtain the initial SR results $I_0^{SR} = S_\theta\ (I^{LR}, k_0)$
12	**while**	$i < t$ **do**
13		Update counter $i = i + 1$;
14		Input predicted kernel and SR result from the previous iteration into C_θ and calculate the error $\Delta k_i = C_\theta(I_{i-1}^{SR}, k_{i-1})$;
15		Update the predicted blur kernel $k_i = k_{i-1} + \Delta k_i$;
16		Update the SR result $I_i^{SR} = S_\theta(I^{LR}, k_i)$;
17	**return**	Output the final SR result I_t^{SR}

Supplementary Note 3. Sample Pre-staining for Laser Microdissection

The protocol of immunohistochemical staining for FFPE sections was consistent with conventional steps[1-3]. Briefly, the tissue section was dewaxed in xylene for 20 min twice, followed by rehydrated in a gradient series of alcohol (100%, 95%, 80%, 70%, 0%) for 5 min each. Subsequently, antigen repair was performed by immersing the sections in preheated citrate buffer (pH= 6.0) in a water bath at 90 degrees Celsius for 30 min. In order to detect the bacteria in the cells, 0.2% Triton X-100 was used to help the antibody enter the cell smoothly. Then, the sections were treated with 3% hydrogen peroxide in PBS for 15 min at room temperature (RT) to quench the activity of endogenous peroxidase. After rinsing with PBS, the sections were blocked with 3% BSA in PBS for 20 min at 37 degrees Celsius. Antibodies targeted to bacterial LPS (1:1000 dilution) or LTA (1:400 dilution) were then added and incubated overnight at 4 degrees Celsius. The following day, a peroxidase-conjugated polymer system was used to detect the presence of G^- or G^+ bacteria. Diaminobenzidine was used as a chromogen, followed by counterstaining of cell nuclei on the sections with hematoxylin to visualize bacterial and cellular expression.

Supplementary Note 4. Laser Microdissection of Bacterial Enrichment Regions

Laser microdissection was carried out on consecutive sections (with film) of the stained sections to collect the bacterial enrichment tissue. The designated tissue area was cut and catapulted into a collection tube cap by a laser spot with appropriate energy (PALM, Zeiss). The collection tube was pre-filled with an appropriate amount of lysis buffer (1.5% SDS; 50mM DTT; 100mM Tris-HCl) on the cap, whose volume would be brought up to 100 µl after cutting completion. The sample was stored at -80 degrees Celsius for future use.

Supplementary Note 5. Label-free Quantitative Proteomics

The sample stored at -80 degrees Celsius from the previous step was thawed in a 37 degrees Celsius water bath, followed by ultrasonication in an ice-water bath for 1 hour. The sample was then placed in a metal bath at 99 degrees Celsius for 1 hour, followed by ultrasonication again in an ice-water bath for 1 hour. The whole protein extract was obtained by collecting the supernatant after centrifuging at 16,000g for 10 min and the protein concentration was measured by a micro-spectrophotometer (Nano-100, ALLSHENG). Then, the supernatant was transferred to a 10 kD ultrafiltration column with additional 250 μl of wash buffer (8M urea; 100mM DTT; 100mM Tris-HCl), followed by centrifugation at 10,000g for 25 min for six times. After that, the sample was incubated with 100 μl of iodoacetamide (IAA) buffer (50 mM IAA; 100 mM Tris-HCl) for 30 min and centrifuged at 10,000g for 10 min. The samples were then washed with wash buffer (200 μl/time for three times) and 50mM bicarbonate (300 μl/time for three times). Subsequently, each sample was incubated with 100 μl of 50mM bicarbonate and trypsin (1:50 dilution) at 37 degrees Celsius overnight, followed by centrifugation with a new collection tube at 12,000g for 20 min. The samples were resuspended in 100 μl of 50mM bicarbonate and centrifuged at 12,000g for 10 min at RT. Then, the ultrafiltration column was removed. Formic acid (FA) was added to the collection tube to a final concentration of 1%, followed by centrifugation at 1,000g for 1 min at RT. For desalination of the sample, a desalting column was activated with 600 μl of methanol (mass spectrometry-grade) and centrifuged at 1,000g for 1 min at RT. The column was equilibrated twice with 200 μl of 0.1% FA and centrifuged at 950g for 1 min at RT. Next, an equal volume of 0.1% FA was added to the sample and loaded onto the desalting column, followed by centrifugation at 950g for 1 min at RT. The column was then rinsed twice with 200 μl of 0.1% FA and centrifuged at 950g for 1 min at RT. For elution, a new collection tube was used, and 100 μl of 60% acetonitrile-0.1% FA was added, followed by centrifugation at 950g for 1 min at RT. The flow-through was then reloaded onto the column and centrifuged at 5,000g for 3 min at RT. Finally, the samples were dried

under vacuum (Eppendorf) and stored at -80 degrees Celsius, which would be redissolved with 0.1% FA to a concentration of 0.5 μg/μL for Liquid Chromatography-tandem Mass Spectrometry (LC-MS/MS).

Sample analysis was performed on an EASY-nLC 1200 system (Thermo Fisher Scientific) coupled to an Orbitrap mass spectrometer (Q Exactive HF-X, Thermo Fisher Scientific). Peptides were loaded to an AcclaimPepMapTM100 C18 trap column (75 μm×2cm,3 μm, Thermo Fisher Scientific) at 2 μL/min with solvent A (0.1% formic acid in water) and eluted with a 120min gradient on an AcclaimPepMapTMRSLCC18 analytical column (75 μm×25 cm, 2 μm, Thermo Fisher Scientific) at a flow rate of 300 nL/min. The gradient elution program was as follows: 0–1min, 1% to 8% solvent B (acetonitrile-water (8:2, v/v) with 0.1% formic acid); 1–98min, 8% to 28% solvent B; 98–112min, 28% to 36% solvent B; 112–116 min, 36%-100% solvent B; 116–120min, 100% solvent B.

Mass Spectrometry parameters were set to: (1) MS: 350–1200 scan range (m/z); 60,000 resolution; 3e6 AGC target; 50ms maximum injection time (MIT); The 20 most intense ions were fragmented by HCD; (2) HCD-MS/MS: 17m/z isolation window; 15,000 resolution; 2e5 AGC target; 25ms MIT; NCE: 28.

Supplementary Note 6. Analysis of label-free proteomics data

A total of 40 samples from 20 patients (2 samples per patient) were analyzed using mass spectrometry (Q Exactive HF-X). Mass spectrometry raw files were searched against the UniProtKB database (UniProt, 2021), then analyzed in Protein Discovery©2.4 (developed by Thermo Fisher Scientific) with default parameters. Up to two missed cleavages were allowed. 1% false discovery rate (FDR) threshold was used in both protein and peptide identifications. Totally, 5,117 proteins were yielded and subsequently utilized for downstream analysis.

In the HER2/ LA/ LB/ TNBC breast cancer samples, 179/ 35/ 52/ 83 proteins were significantly up-regulated and 27/ 40/ 20/ 26 proteins were significantly down-regulated in the bacterial-enrichment region relative to the bacterial non-enrichment region. There were 9/ 6/ 6/ 5/ 3/ 3 significantly different proteins expressed in TNBC and HER2/ LB and TNBC/ LB and HER2/ LA and HER2/ LA and LB/ LA and TNBC. In HER2 breast cancer samples, 7 proteins significantly up-regulated in bacterial enrichment regions were associated with positive immune function. In LA breast cancer samples, 2 proteins significantly down-regulated in bacterial enrichment regions were associated with negative immune function. Volcano and Venn diagrams were drawn using the OmicStudio tools[4] at https://www.omicstudio.cn/tool.

Significant difference proteins were entered into the website of Metascape[5] for Gene Ontology analysis and Kyoto Encyclopedia of Genes and Genomes analysis. As for protein interaction network, input significant difference proteins into STRING website to get comprehensive results.

Supplementary Note 7. Calculate the accuracy of cell extraction

We used the fluorescent/metal-dual labeling approach to obtain both IMC images, and confocal images with the same underlying scene as the IMC images. Because confocal image had higher PSNR and resolution, we used it as the ground truth to evaluate performance.

The confocal images were fed into the Cellpose program running with default parameters to obtain preliminary cell segmentation results. The default parameter settings according to the literature[11] were shown in the Supplementary Table 10. Then the expertise researcher adjusted the Cellpose program parameters to obtain the accurate segmentation results as the ground truth of cell segmentation. When the raw IMC and SpiDe-Sr enhanced IMC were fed into the Cellposs program, for the control variable, the program was only run with the default parameters, and the parameters were no longer manually adjusted. It should be noted that the output of Cellpose was only the mask of cell segmentation of image. And whether the cells were accurately extracted, or missed or extra extracted, should be manually counted by the researcher. Specifically, in the SpiDe-Sr performance validation phase, we used Cellpose for cell segmentation of confocal and IMC images. The confocal images were input into the Cellpose program and run with default parameters (Supplementary Table 10) to obtain preliminary cell segmentation results. The two parameters, namely cell diameter (pixels) and model zoo, were then readjusted. The calibrated cell diameter was a numerical value that could be manually readjusted for specific conditions. The model zoo was set to cytoplasm pattern (cyto). As for IMC images, in order to avoid adding artificial bias as much as possible, we did not manually correct the 2 parameters again. In the stage of breast cancer microenvironment analysis, we first used the default parameters for cell segmentation of breast cancer images. The model zoo was set to cytoplasm pattern (cyto). And then we readjusted the value of approximate cell diameter one by one in the user interface to make the segmentation as accurate as possible.

Supplementary Tables

Supplementary Table 1. Antibody Information

Antibody	Mental Tag	Clone	Vendor	Catalog number	Concentration (μg/ml)
Tubulin	/	/	Beyotime	AF1216	20
CD45	/	/	Beyotime	AF7839	20
CD34	/	/	Beyotime	AF1387	20
Secondary antibody	^{165}Ho	Poly4064	Biolegend	406416	10
PKCD	^{144}Nd	EPR17075	Abcam	ab222229	5
PR	^{145}Nd	YR85	Abcam	ab206926	1
IFI6	^{147}Sm	/	Abcam	ab192314	5
HER2	^{148}Nd	EP1045Y	Abcam	ab194979	0.25
Ki67	^{152}Sm	SP6	Abcam	ab197547	0.15
ISG15	^{153}Eu	/	Abcam	ab285370	5
ER	^{163}Dy	EPR4097	Abcam	ab167610	3
ZC3HAV1	^{168}Er	/	Abcam	ab154680	5
LPS	^{165}Ho	WN1 222-5	HycultBiotech	HM6011	1
LTA	^{169}Tm	mAb 55	HycultBiotech	HM2048	0.5
CD45	^{141}Pr	HI30	Biolegend	304045	0.15
CD68	^{174}Yb	/	Abcam	ab283667	5
CD8a	^{175}Lu	CAL66	Abcam	ab251596	1
CD19	^{176}Yb	6OMP31	Invitrogen	14-0194-95	2.5

Supplementary Table 2. Reagent Information

Regent	Catalog	Vendor	Region
Dulbecco's modified Eagle's medium	SH30243.01	HyClone	USA
fetal bovine serum	10099141C	Gibco	USA
penicillin-streptomycin	15140163	Gibco	USA
positively charged slides	188105W	CITOTEST	Jiangsu, China
PBS	B540627	Sangon Biotech	Shanghai, China
paraformaldehyde	C104188-100g	Aladdin	Shanghai, China
sucrose	10021418	Sinopharm	Beijing, China
OCT	6502,	Thermo Fisher scientific	USA
Maxpar antibody labelling kit	201165A,	Fluidigm Sciences	USA
protein stabilizing cocktail	89806	Thermo Fisher scientific	USA
xylene	A69925	Innochem	Beijing, China
ethanol	100092680	Sinopharm	Beijing, China
Triton X-100	A110694-0100	Sangon Biotech	Shanghai, China
BSA	B09354	Innochem	Beijing, China
DAPI	D9542	Sigma-Aldrich	USA
191Ir/ 193Ir DNA intercalator	201192a	Fluidigm Sciences	USA
hydrogen peroxide	A001847	Sangon Biotech	Shanghai, China
hematoxylin	C0105S	Beyotime	Shanghai, China
urea	B20910	yuanye	Shanghai, China
SDS	R21371	yuanye	Shanghai, China
DTT	ST043	Beyotime	Shanghai, China
IAA	B21810	yuanye	Shanghai, China
methanol	R40121	Thermo Fisher scientific	USA
trypsin	VA9000	Promega	USA
FA	28905	Thermo Fisher scientific	USA

Supplementary Table 3. Program Operating Environment

Important Package	Version
python	3.7.11
tensorflow	1.15.0
torch	1.9.1+cu111
torchvision	0.10.1+cu111
numpy	1.20.3
scipy	1.7.2
sklearn	0.0
scanpy	1.8.2
tensorflow-estimator	2.6.0
yaml	0.2.5
opencv-python	4.5.4.58
pandas	1.3.4
imageio	2.12.0
scikit-image	0.19.3

Supplementary Table 4. Significantly different proteins (Top 25) in bacterial enrichment regions of HER2 breast cancer samples

Accession	Description	Average expression in enrichment regions	Average expression in non-enrichment regions
Q9NQT8	Kinesin-like protein KIF13B	87.075	27.925
Q9NVV4	Poly(A) RNA polymerase, mitochondrial	197.225	98.95
Q9P0S3	ORM1-like protein 1	317	192.1
P36551	Oxygen-dependent coproporphyrinogen-III oxidase, mitochondrial	82.175	186.65
P02745	Complement C1q subcomponent subunit	90.725	160.825
Q96L21	60S ribosomal protein L10-like	86.35	284.4
O00142	Thymidine kinase 2, mitochondrial	492.475	137.1
Q6ZMG9	Ceramide synthase 6	280.6	126.8
Q9Y6A9	Signal peptidase complex subunit 1	263.325	146.85
Q969U7	Proteasome assembly chaperone 2	217.25	79.3
O15160	DNA-directed RNA polymerases I and III subunit RPAC1	189	56.975
Q3ZAQ7	Vacuolar ATPase assembly integral membrane protein VMA21	140.575	73.225
Q9BY43	Charged multivesicular body protein 4a	321.825	121.2
Q14894	Ketimine reductase mu-crystallin	165.025	81.375
Q9UMX5	Neudesin	174.4	101.675
Q99447	Ethanolamine-phosphate cytidylyltransferase	251.75	110.04
Q8NCL4	Polypeptide N-acetylgalactosaminyltransferase 6	516.525	133.375
O76015	Keratin, type I cuticular Ha8	137.3	86.95
Q7L5N7	Lysophosphatidylcholine acyltransferase 2	134.025	77.975
Q02318	Sterol 26-hydroxylase, mitochondrial	160.6	84.45
Q96EE3	Nucleoporin SEH1	189.075	103.575
Q15382	GTP-binding protein Rheb	416.925	183.675
O15400	Syntaxin-7	175.98	48.02
Q969E4	Transcription elongation factor A protein-like 3	62.6	27.92
Q8NFU3	Thiosulfate:glutathione sulfurtransferase	86.06	32.08

Supplementary Table 5. Significantly different proteins (Top 25) in bacterial enrichment regions of LA breast cancer samples

Accession	Description	Average expression in enrichment regions	Average expression in non-enrichment regions
Q9P206	Uncharacterized protein KIAA1522	219.05	440.825
Q86W10	Cytochrome P450 4Z1	51.64	109.9
Q15154	Pericentriolar material 1 protein	194.55	326.925
P07476	Involucrin	70.925	247.325
O95232	Luc7-like protein 3	146.425	400
Q13546	Receptor-interacting serine/threonine-protein kinase 1	77.8	147.375
Q9Y679	Lipid droplet-regulating VLDL assembly factor AUP1	36.75	61.525
Q86TJ2	Transcriptional adapter 2-beta	156	361.575
P51531	Probable global transcription activator SNF2L2	110.86	211.1
Q27J81	Inverted formin-2	43.16	119.425
Q99572	P2X purinoceptor 7	164.1	92.7
O43291	Kunitz-type protease inhibitor 2	75.525	21.925
Q00587	Cdc42 effector protein 1	181.525	61.875
Q86TD4	Sarcalumenin	52.525	279.675
Q92619	Rho GTPase-activating protein 45	60.94	26.38
P49756	RNA-binding protein 25	76.175	119.525
O75822	Eukaryotic translation initiation factor 3 subunit J	98.24	53.74
Q9NTX5	Ethylmalonyl-CoA decarboxylase	26.1	66.675
O60656	UDP-glucuronosyltransferase 1A9	85.05	46.2
Q96EY8	Corrinoid adenosyltransferase	122.9	207.875
Q03169	Tumor necrosis factor alpha-induced protein 2	93.875	25.175
P02679	Fibrinogen gamma chain	54.5	38.14
Q6P1A2	Lysophospholipid acyltransferase 5	44.24	138.1
Q12907	Vesicular integral-membrane protein VIP36	95.16	51.76
Q9C075	Keratin, type I cytoskeletal 23	96.12	140.22

Supplementary Table 6. Significantly different proteins (Top 25) in bacterial enrichment regions of LB breast cancer samples

Accession	Description	Average expression in enrichment regions	Average expression in non-enrichment regions
O95340	Bifunctional 3'-phosphoadenosine 5'-phosphosulfate synthase 2	47.475	18.96
P45877	Peptidyl-prolyl cis-trans isomerase C	92.5	50.45
Q03692	Collagen alpha-1(X) chain	62.725	21.225
Q7L5D6	Golgi to ER traffic protein 4 homolog	108.35	45.975
P36507	Dual specificity mitogen-activated protein kinase kinase 2	113.05	33.1
P40616	ADP-ribosylation factor-like protein 1	118.38	26.26
P36952	Serpin B5	48	20.92
Q9BRJ7	Tudor-interacting repair regulator protein	199.475	61.125
O43819	Protein SCO2 homolog, mitochondrial	48.925	175.1
O15321	Transmembrane 9 superfamily member 1	46.05	14.175
P05166	Propionyl-CoA carboxylase beta chain, mitochondrial	148.275	58.7
O43688	Phospholipid phosphatase 2	171.525	84.6
Q8IYQ7	Threonine synthase-like 1	77.95	26.575
Q9UBX7	Kallikrein-11	326.675	182.125
P49863	Granzyme K	244.775	121.575
Q9UI10	Translation initiation factor eIF-2B subunit delta	112.075	30
O14880	Microsomal glutathione S-transferase 3	208.55	69
Q7LG56	Ribonucleoside-diphosphate reductase subunit M2 B	79.26	151.225
Q8WUW1	Protein BRICK1	135.3	60.225
Q92817	Envoplakin	128.1	72.075
P51531	Probable global transcription activator SNF2L2	143.25	276.9
Q9Y5L2	Hypoxia-inducible lipid droplet-associated protein	67.02	138.42
Q8NCN5	Pyruvate dehydrogenase phosphatase regulatory subunit, mitochondrial	45.7	133.075
Q6E0U4	Dermokine	142.9	272.825
Q9BUL8	Programmed cell death protein 10	58.55	77.825

Supplementary Table 7. Significantly different proteins (Top 25) in bacterial enrichment regions of TNBC breast cancer samples

Accession	Description	Average expression in enrichment regions	Average expression in non-enrichment regions
P46939	Utrophin	64.26666667	205.3333333
Q96HR9	Receptor expression-enhancing protein 6	24.23333333	44.76666667
Q9UJZ1	Stomatin-like protein 2, mitochondrial	188.25	45.5
Q93009	Ubiquitin carboxyl-terminal hydrolase 7	184.025	71.925
P35813	Protein phosphatase 1A	125.8666667	57.36666667
Q12884	Prolyl endopeptidase FAP	150	56.43333333
P53680	AP-2 complex subunit sigma	170.675	82.7
P47736	Rap1 GTPase-activating protein 1	75.76666667	32.76666667
P17655	Calpain-2 catalytic subunit	97.825	51.175
Q9Y3D9	28S ribosomal protein S23, mitochondrial	149.35	33.3
Q9GZN8	UPF0687 protein C20orf27	220.175	68.4
Q9C030	Tripartite motif-containing protein 6	164.675	61.1
O75821	Eukaryotic translation initiation factor 3 subunit G	156.875	83.23333333
P51159	Ras-related protein Rab-27A	211.25	83.76666667
P61803	Dolichyl-diphosphooligosaccharide--protein glycosyltransferase subunit DAD1	166.8	78.075
Q01105	Protein SET	169.025	97.975
Q969H8	Myeloid-derived growth factor	144	65.175
P35637	RNA-binding protein FUS	227.775	103.275
Q8WVV9	Heterogeneous nuclear ribonucleoprotein L-like	312.1666667	186.6
Q9NQT8	Kinesin-like protein KIF13B	144.3333333	77.96666667
Q14108	Lysosome membrane protein 2	95	27.85
P21926	CD9 antigen	230	99.275
P22105	Tenascin-X	51.75	135.875
O60701	UDP-glucose 6-dehydrogenase	76.15	51.875
P82909	28S ribosomal protein S36, mitochondrial	85.2	198.8

Supplementary Table 8. Correlation between LPS expression and other biomarker expressions

Sub-types of breast cancer	Positively associated with LPS (P> 0.50)	Negatively associated with LPS (P> 0.50)
HER2	ISG15, IFI6, HER2, ZC3HAV1	CD45, CD68
LA	IFI6, PKCD, CD19, ZC3HAV1, ISG15	\
LB	IFI6	CD68
TNBC	CD19, CD45, PKCD, ZC3HAV1, ISG15	CD8a

(P, Pearson correlation coefficient)

Supplementary Table 9. Correlation between LTA expression and other biomarker expressions

Sub-types of breast cancer	Positively associated with LTA (P> 0.75)	Negatively associated with LTA (P> 0.75)
HER2	CD45, PR, CD8a, Ki67	HER2, LPS
LA	CD68, LPS, IFI6, ZC3HAV1	Ki67, PR
LB	LPS	\
TNBC	ER, ISG15, PR, CD45, IFI6	\

(P, Pearson correlation coefficient)

Supplementary Table 10. Default parameters for Cellpose

Parameters	Settings
Up/down or W/S	RGB
page up/down	image
brush size	3
MASK ON	Yes
single stroke	Yes
outlines on	No
scale disk on	Yes
use GPU	Yes
flow_threshold	0.7
cellprob_threshold	0.0
stitch_threshold	0.0
cell diameter (pixels)	calibrate
model zoo	default

Supplementary Figures

Supplementary Fig. 1

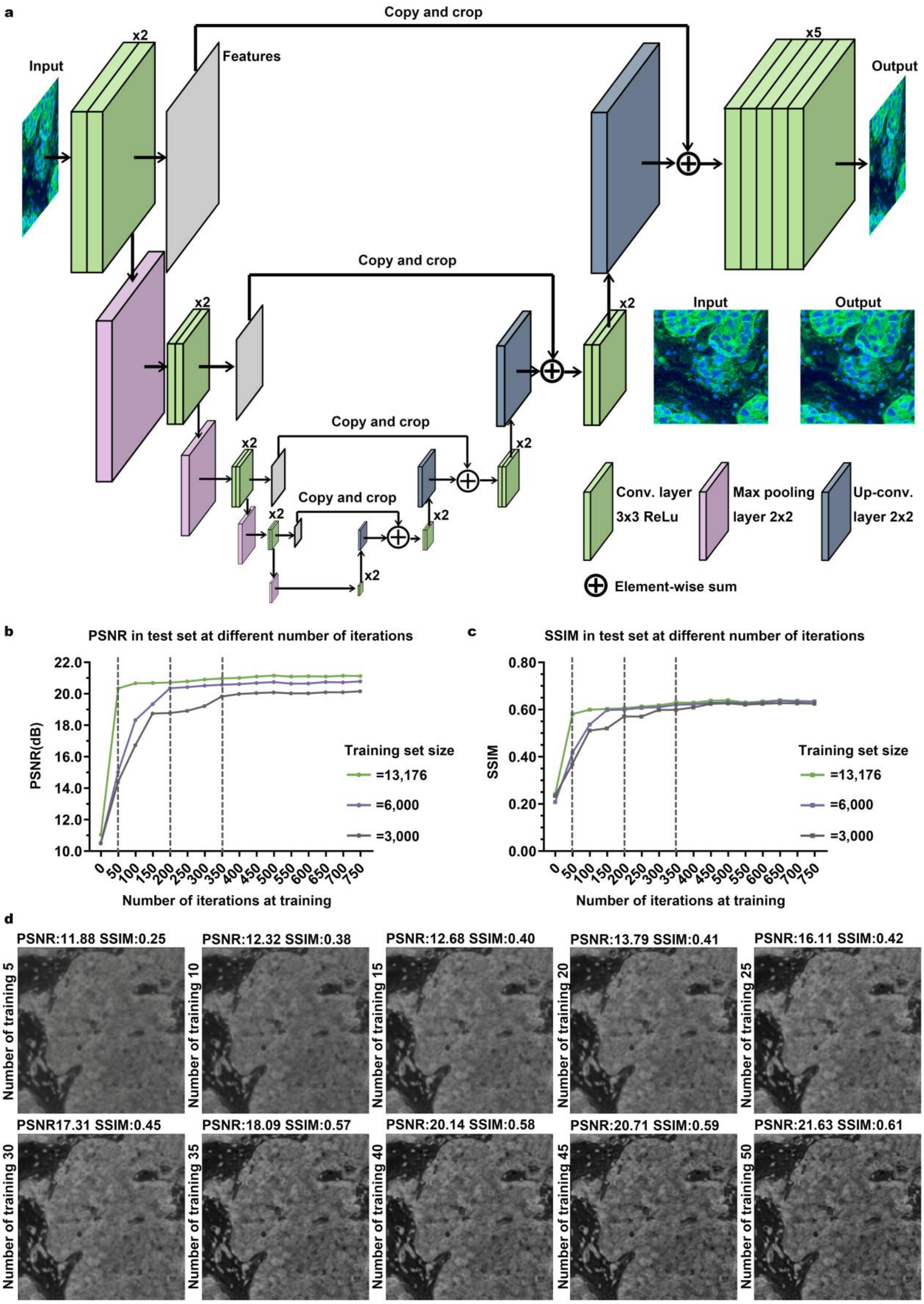

Supplementary Fig. 1 The architecture of the $\boldsymbol{U_\theta}$ and training process of denoising module. a, The architecture of the U_θ. b-c, The PSNR (b) and SSIM (c) of the validation set for models trained on different training set sizes with different number of trainings. The green/ purple/ gray line indicated the result for a training set size of 13,176/ 6,000/ 3,000. Dashed line indicated the number of iterations when the model converged. When the training set size was 13,176/ 6,000/ 3,000, the model was trained 350/ 200/ 50 times to converge. The model trained with a training set size of 13,176 images was able to obtain images with maximum PSNR and SSIM. d, Output of denoising module for different number of trainings before convergence (training set size= 13,176). The Fig. 1d showed that with the increase in the number of model training, the noise of the output image gradually decreased, and the PSNR and SSIM of the output image were gradually improved. The cells in the image were getting clearer and clearer. Abbreviations and remarks: PSNR, peak signal-to-noise ratio, larger means less noise. SSIM, structural similarity, larger means more similar to the ground truth.

Supplementary Fig. 2

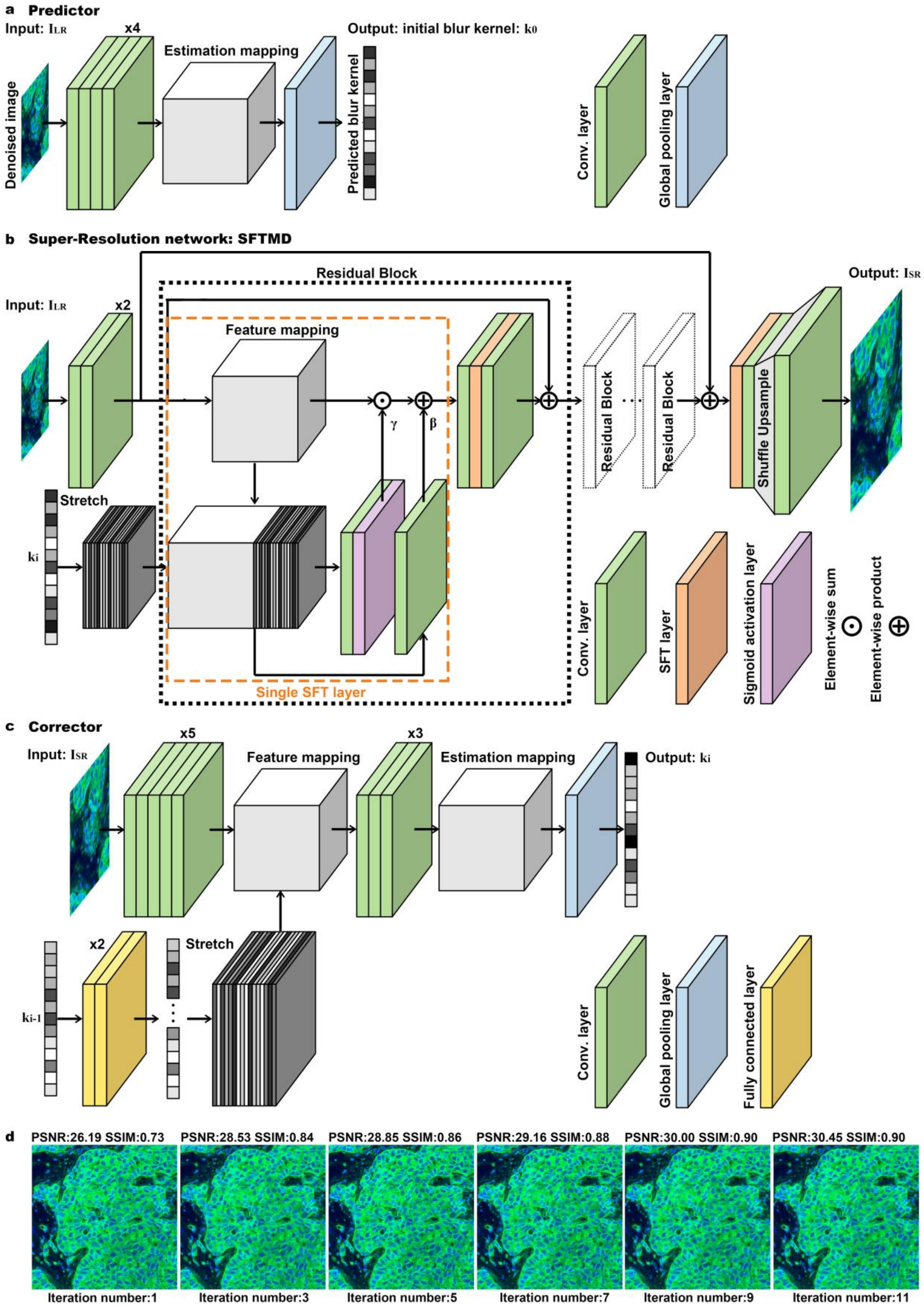

Supplementary Fig. 2 The architecture of the super-resolution (SR) module. a-c, The architecture of (a) the blur kernel predictor (P_{θ}), (b) the SR network (S_{θ}) and (c) the blur kernel corrector (C_{θ}). d, Output of SR module at different number of iterations. The number of iterations corresponded to the number of blur kernel corrections. We could see that with the increase in the number of the blur kernel corrections, the cells in the image output by the super-resolution model became clearer and clearer.

Supplementary Fig. 3

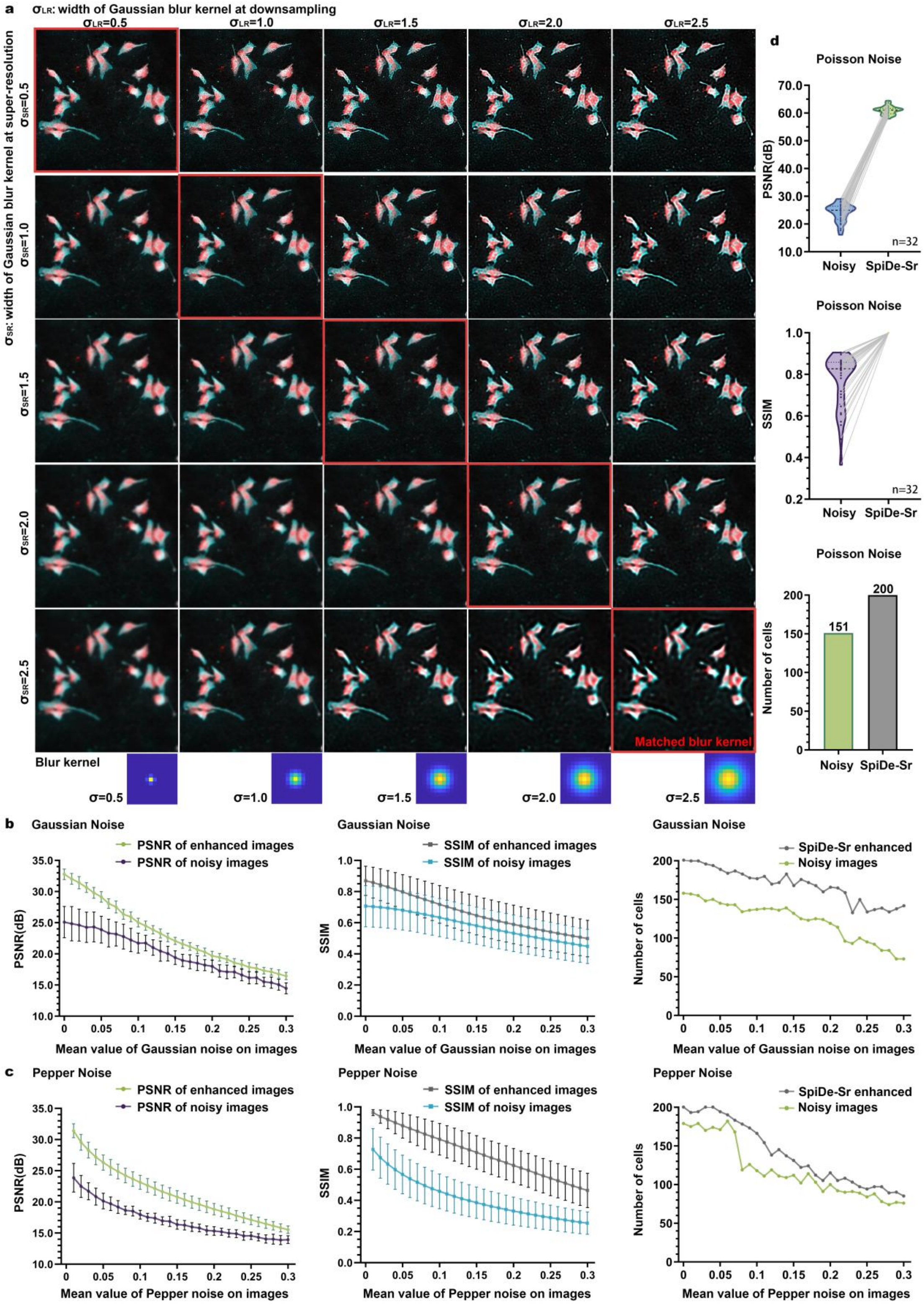

Supplementary Fig. 3 The sensitivity of super-resolution to blur kernel mismatch and test results for different noisy scenarios. a, The sensitivity of super-resolution to blur kernel mismatch. The σ_{LR} was the width of the blur kernel for down-sampling and the σ_{SR} was the width of the blur kernel for up-sampling. The images in the red boxes had the same blur kernels for up-sampling (super-resolution) as for down-sampling. We could see that the images were best restored when the up-sampling and down-sampling blur kernels matched. Iteratively adjusting the blur kernel could avoid the gap between the predicted blur kernel and the real blur kernel to be too large. b-c, The PSNR, SSIM and the number of extracted cells of test set with different Gaussian (b)/ pepper (c) noise levels before and after the SpiDe-Sr. d, The PSNR, SSIM and the number of extracted cells of test set with Poisson noise. We could see whether the images were superimposed with Gaussian noise, Peper noise, or Poisson noise, the SpiDe-Sr could effectively optimize the images to improve the PSNR and SSIM of the images, as well as the accuracy of the subsequent cell extraction. The improvement of the PSNR and SSIM of the images, and cell extraction accuracy were statistically significant (two sided paired-samples *t*-test, $P<0.001$).

Supplementary Fig. 4

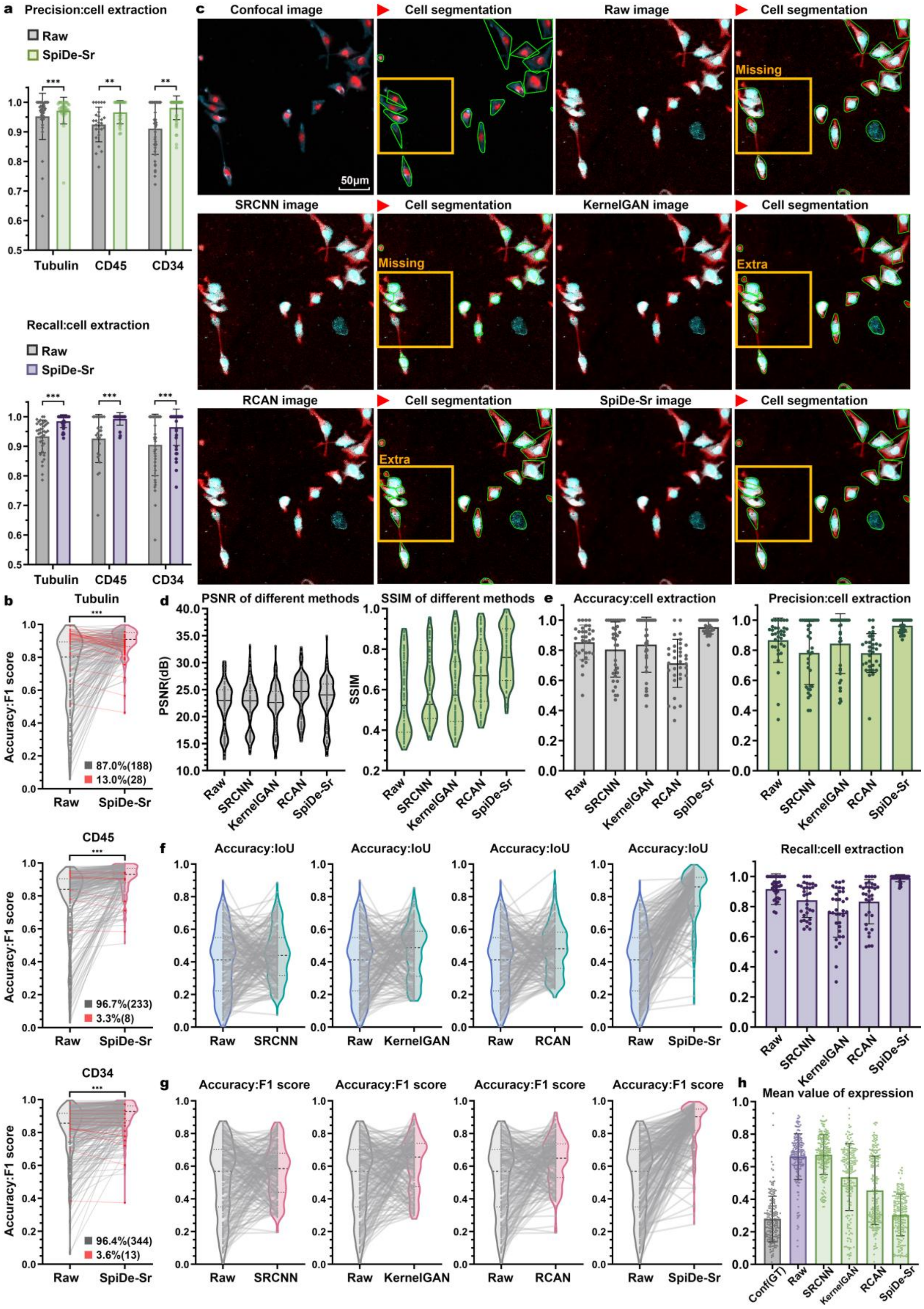

Supplementary Fig. 4 Validation of different SR methods (SRCNN/ KernelGAN/ RCAN/ SpiDe-Sr) on IMC images of cells. a, The precision and recall of cell extraction in images for Tubulin/ CD45/ CD34 being labeled. n=52 (Tubulin)/ 36 (CD45)/ 71 (CD34) images. The precision and recall were both complementary to accuracy, in order to fully illustrate that SpiDe-Sr improved the accuracy of cell extraction in cell IMC images. (two-sided *t*-test, ***P*< 0.01, ****P*< 0.001). b, Violin-scatter plots showed the distribution of F_1 score of accurately extracted cells in IMC images before and after SpiDe-Sr enhancement vs. extracted cells in GT images. Each line represented one of 216/ 241/ 357 cells. Increasing pairs were colored gray and decreasing pairs were colored red. The F_1 score was complementary to the IoU, in order to fully illustrate that cell boundaries were segmented more accurately (two-sided paired-samples *t*-test, ****P*< 0.001). c, Images before and after SRCNN/ KernelGAN/ RCAN/ SpiDe-Sr enhancement and the corresponding cell segmentation. The confocal images (20x) were treated as ground truth (GT). Individual cell boundaries were colored green. The SpiDe-Sr enhanced image was visually better and cell segmentation in it was more accurate compared to the other three methods. d, The PSNR and SSIM comparisons of the four SR methods with the GT images. Each point indicated one of 159 (The sum of the number of images labeled by the three biomarkers) images. e, The accuracy (gray), precision (green) and recall (purple) of cell extraction after enhancement by four SR methods. n= 159 images. f-g, The distribution of IoU (f) and F_1-score (g) of accurately extracted cells in IMC images before and after enhancement of four SR methods vs. extracted cells in GT images. Each line represented one of n= 202 cells. h, The normalized marker expression in accurately extracted cells before and after enhancement of four SR methods. The ground truth was colored in gray.

Supplementary Fig. 5

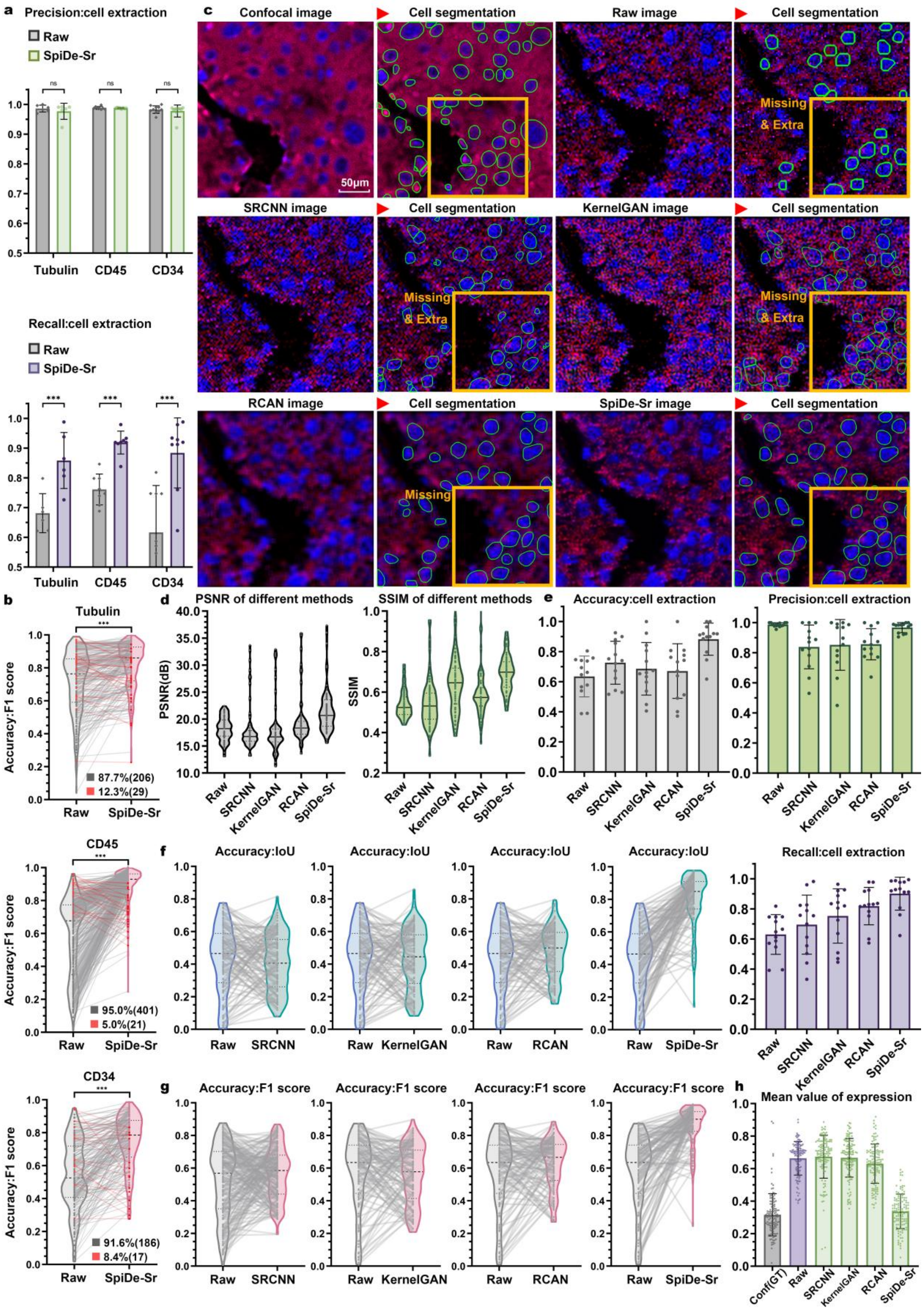

Supplementary Fig. 5 Validation of different SR methods (SRCNN/ KernelGAN/ RCAN/ SpiDe-Sr) on IMC images of mouse fatty liver tissues. a, The histogram-scatter plots illustrated the precision (above) and recall (below) of cell extraction in IMC images for Tubulin/ CD45/ CD34 being labeled. The precision and recall were both complementary to accuracy, in order to fully illustrate that SpiDe-Sr improved the accuracy of cell extraction in cell IMC images. (two sided *t*-test, $^{**}P< 0.01$, $^{***}P< 0.001$). b, The violin-scatter plots illustrated the F_1 score distribution of accurately extracted cells in IMC images before and after SpiDe-Sr enhancement. n= 235/ 422/ 203 cells. The F_1 score was complementary to the IoU, in order to fully illustrate that cell boundaries were segmented more accurately (two sided paired-samples *t*-test, $^{***}P< 0.001$). c, Images before and after enhancement of SRCNN/ KernelGAN/ RCAN/ SpiDe-Sr and the corresponding cell segmentation. The SpiDe-Sr enhanced image had the best visual performance and the least amount of extra and missed extraction of cells in the image. d, The violin-scatter plots showed the PSNR (gray) and SSIM (green) before and after enhancement by four methods. n= 75 images. e, The histogram-scatter plots showed the accuracy (gray), the precision (green) and the recall (purple) of cell extraction before and after enhancement by four methods. f-g, The violin-scatter plots illustrated the IoU (f) and the F_1 score (g) distribution of accurately extracted cells in IMC images before and after enhancement by four methods. n= 127 cells. h, The histogram-scatter plots illustrated the normalized marker expression in accurately extracted cells before and after enhancement by four methods.

Supplementary Fig. 6

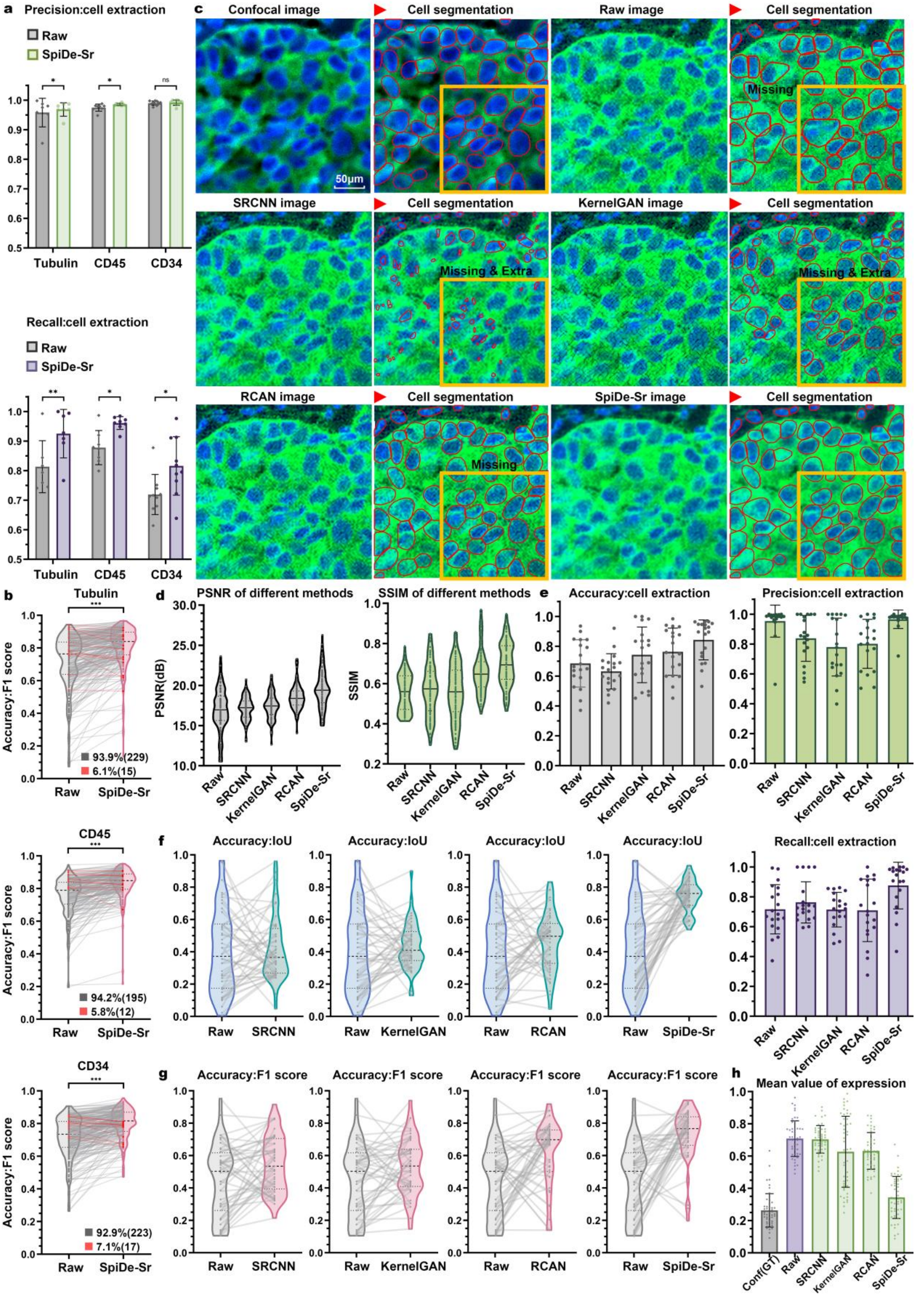

Supplementary Fig. 6 Validation of different SR methods (SRCNN/ KernelGAN/ RCAN/ SpiDe-Sr) on IMC images of human breast cancer tissues. a, The precision and recall of cell extraction in images for Tubulin/ CD45/ CD34 being labeled before and after enhancement of SpiDe-Sr. The precision and recall were both complementary to accuracy, in order to fully illustrate that SpiDe-Sr improved the accuracy of cell extraction in cell IMC images. (two sided *t*-test, **P*< 0.05, ***P*< 0.01). b, The F_1 score distribution of accurately extracted cells in images of Tubulin/ CD45/ CD34 being labeled before and after SpiDe-Sr enhancement. n= 244/ 207/ 240 cells. The F_1 score was complementary to the IoU, in order to fully illustrate that cell boundaries were segmented more accurately (two sided paired-samples *t*-test, ****P*< 0.001). c, Confocal images (considered as GT in our work) and IMC images before and after enhancement by SRCNN/ KernelGAN/ RCAN/ SpiDe-Sr, and the corresponding cell segmentation. d-e, Quantitative evaluation (PSNR (d, gray)/ SSIM (d, green)) of image quality and the accuracy (e, gray)/ the precision (e, green)/ the recall (e, purple) of cell extraction in images before and after the enhancement of the four SR methods. n= 104 images. f-g, Quantitative evaluation of the accuracy of cell boundary detection (f, IoU, g, F_1 score) in images before and after enhancement by four SR methods. n= 48 cells. h, The normalized marker expression in accurately extracted cells in images before and after enhancement by four SR methods.

Supplementary Fig. 7

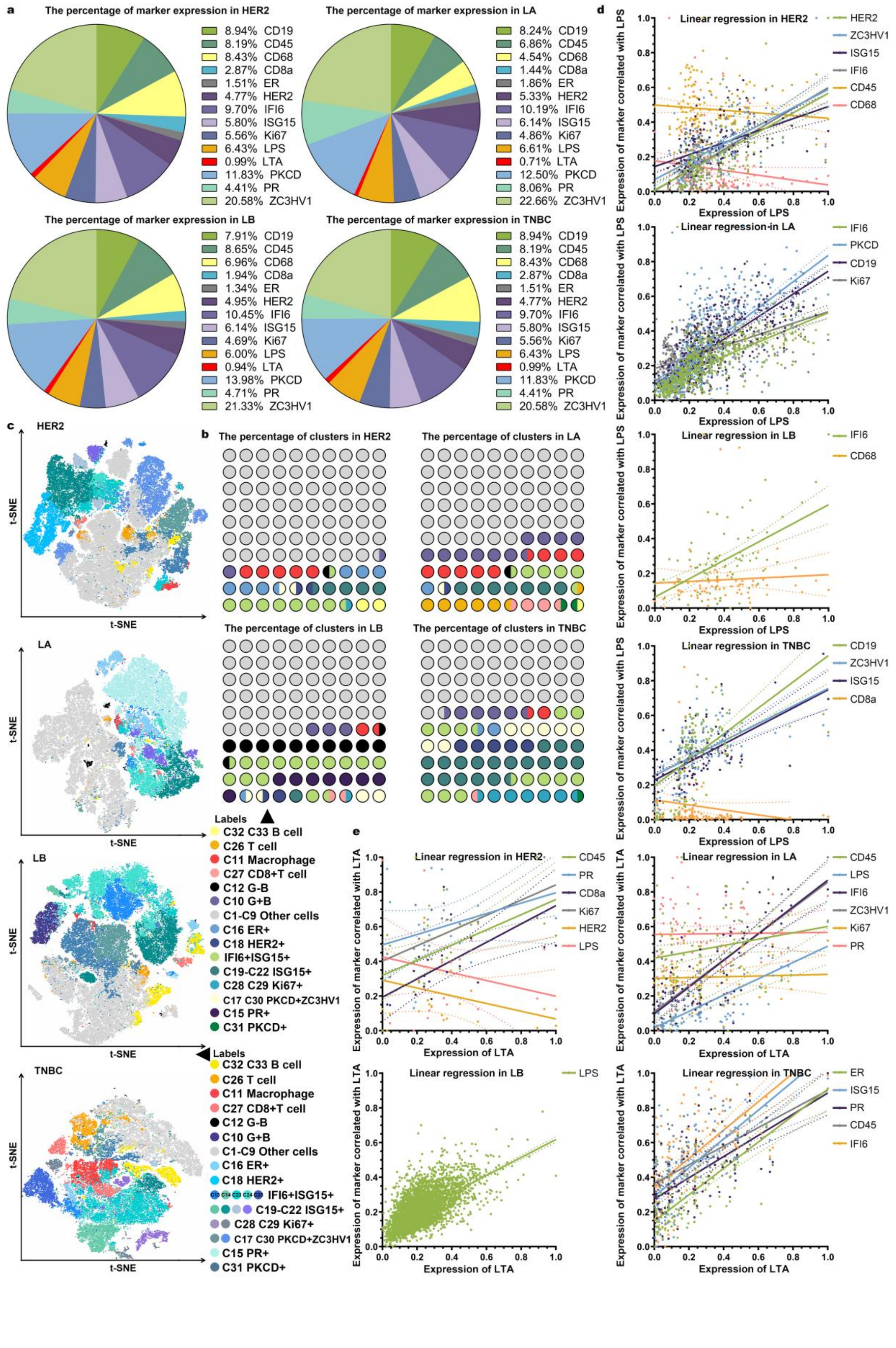

Supplementary Fig. 7 Application of SpiDe-Sr to spatial proteomics data of four major subtypes of breast cancer patients. a-b, The percentage of expression of 14 markers (a) and the percentage of cell number in 33 clusters (b) in four breast cancer subtypes. The two panels were shown to illustrate the data (biomarker expressions and cell clustering result) in detail. c, Map using t-distributed stochastic neighbor embedding (t-SNE) of 86,968 (HER2)/ 55,496 (LA)/ 73,161 (LB)/ 53,931 (TNBC) sub-sampled single cells from high-dimensional images of breast tumors colored by cell-type metacluster identifier. The gray dots were normal cells and the blue-tinted dots were breast cancer cells. Normal and breast cancer cells were well distinguished in the data for all four breast cancer subtypes. Immune cells (B cells were colored in yellow, T cells were colored in orange, macrophages were colored in red, and CD8+ T cells were colored in pink.) were generally distributed between normal cells and breast cancer cells. d-e, The regression analysis between LPS (d)/ LTA (e) and markers positively or negatively correlated with LPS/ LTA in four subtypes of breast tumors. Specific biomarkers were listed in Supplementary Table 8 and Supplementary Table 9.

Supplementary Fig. 8

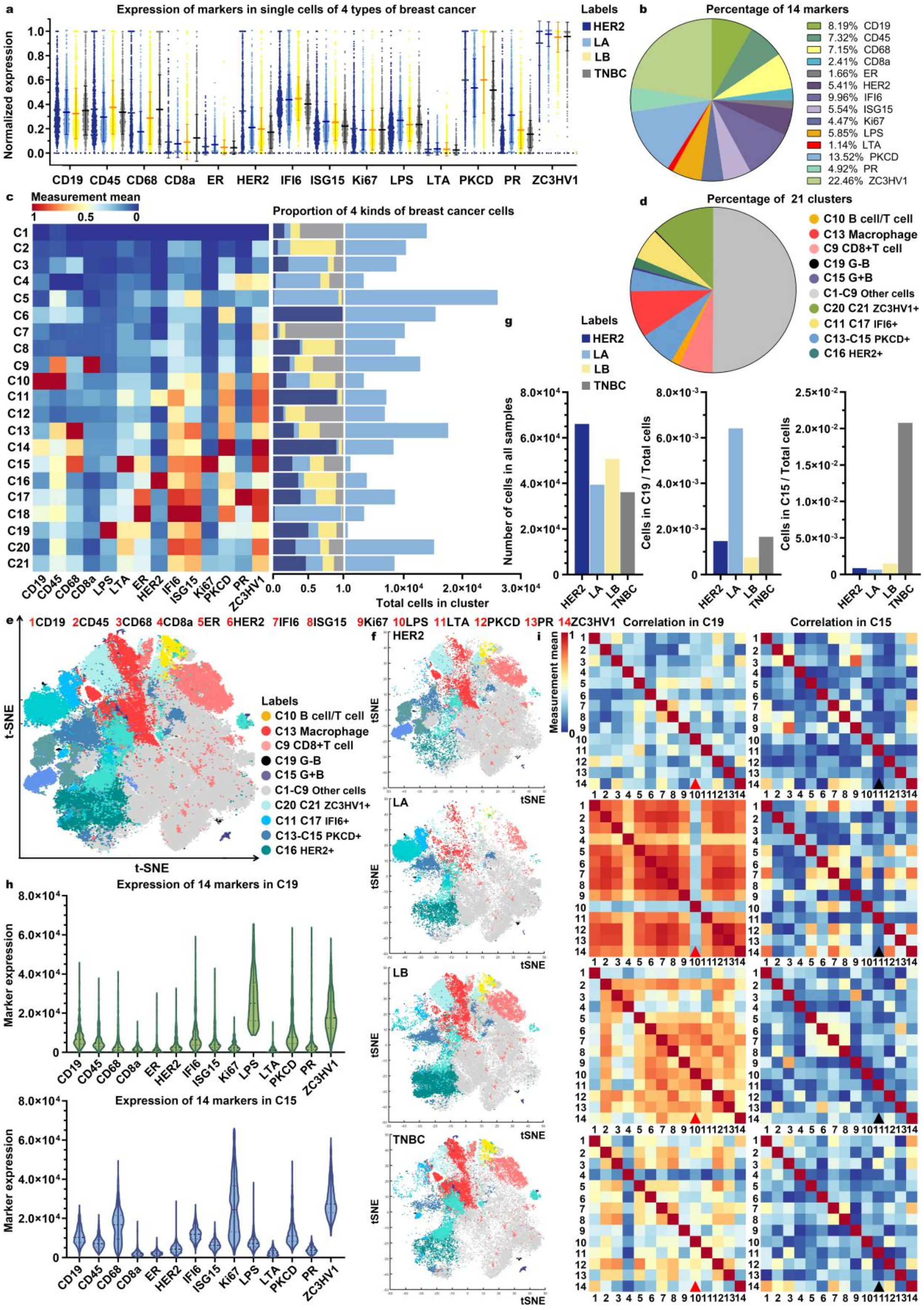

Supplementary Fig. 8 Analysis of raw spatial proteomics data (without SpiDe-Sr enhancement) from patients with four major subtypes of breast tumor. a, Normalized expression of 14 markers in single cells of four breast cancers. Each point indicated one of 6,611 (HER2)/ 3,941 (LA)/ 5,075 (LB)/ 3,618 (TNBC) cells. b, The percentage of each marker expression among all marker expressions. This panel was designed to specify the expression of 14 biomarkers. c, (left) Heat map showing normalized mean marker expression for each PhenoGraph cluster. (middle) Proportion of four subtypes of breast tumor cells in each cluster. (right) The absolute cell counts of each cluster. d, The proportion of cells in each of the 21 clusters relative to the total number of cells. Without SpiDe-Sr enhancement, B cells and T cells could not be distinguished and only 4 tumor cell clusters were identified based on the same IMC dataset because of noise interference or insufficiently precise details. e, Map using t-SNE of 192,445 (all) sub-sampled single cells from high-dimensional images of breast tumors colored by cell-type metacluster identifier. In the panel, normal cells and breast cancer cells could be distinguished, but it was clear that normal cells and immune cells were not well distinguished. f, Map using *t*-SNE of 66,110 (HER2)/ 39,411 (LA)/ 50,746 (LB)/ 36,178 (TNBC) sub-sampled single cells from high-dimensional images of breast tumors colored by cell-type metacluster identifier. g, (left) Number of cells extracted in all breast cancer samples. (middle) The proportion of HER2/ LA/ LB/ TNBC cells in C19 with the highest expression of G^- bacteria marker to the total number of HER2/ LA/ LB/ TNBC cells. (right) The proportion of HER2/ LA /LB/ TNBC cells in C15 with the highest expression of G^+ bacteria marker to the total number of HER2/ LA/ LB/ TNBC cells. h, The absolute expression of 14 markers in cells of (above) C19 and (below) C15. i, Heat map showing the Pearson correlation coefficients of the 14 markers in (left) C19 and (right) C15 with each other. There was no indication that G- or G+ bacteria had any particular correlation in the breast cancer microenvironment.

Supplementary Fig. 9

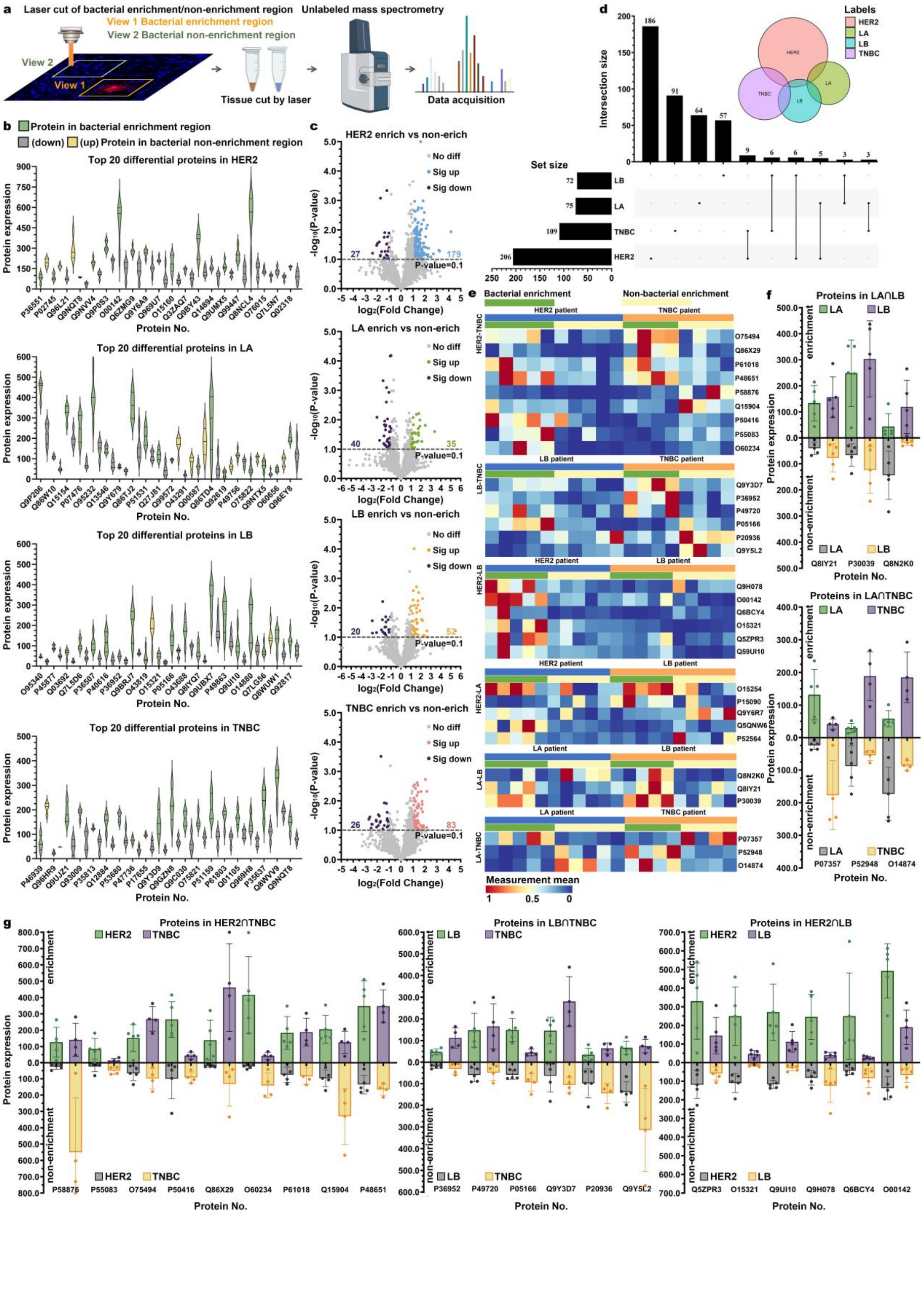

Supplementary Fig. 9 Acquisition and analysis of label-free proteomics with clinical breast cancer specimens. a, Acquisition of label-free proteomics data. b, The 20 proteins with the greatest differences in expression between bacterial enrichment and bacterial non-enrichment tissues in HER2/ LA/ LB/ TNBC. Proteins in bacterial enrichment region were colored green. In bacterial non-enrichment regions, proteins significantly up-regulated were colored yellow and significantly down-regulated proteins were colored gray. c, The volcano plots showed the proteins significantly up- and down-regulated in the bacterial enrichment region compared to the non-enrichment region in four subtypes of breast tumor. In the bacterial-enrichment region, the expressions of 27/ 40/ 20/ 26 (in HER2/ LA/ LB/ TNBC) proteins were significantly increased and 179/ 35/ 52/ 83 (in HER2/ LA/ LB/ TNBC) proteins were significantly decreased. d, Venn plots showed co-expression of significantly different proteins in four subtypes of breast tumor. e, Normalized expression of co-expressed proteins. f-g, Absolute expression of co-expressed proteins in each patient sample. The upper half of the y-axis was for protein expression in bacterial enrichment regions, and the lower half of the y-axis was for protein expression in bacterial non-enrichment regions. This figure was mainly to illustrate the label-free proteomic data in detail. Proteins with significantly increased or significantly decreased expression compared to non-bacterial enrichment regions were visualized.

Supplementary Fig. 10

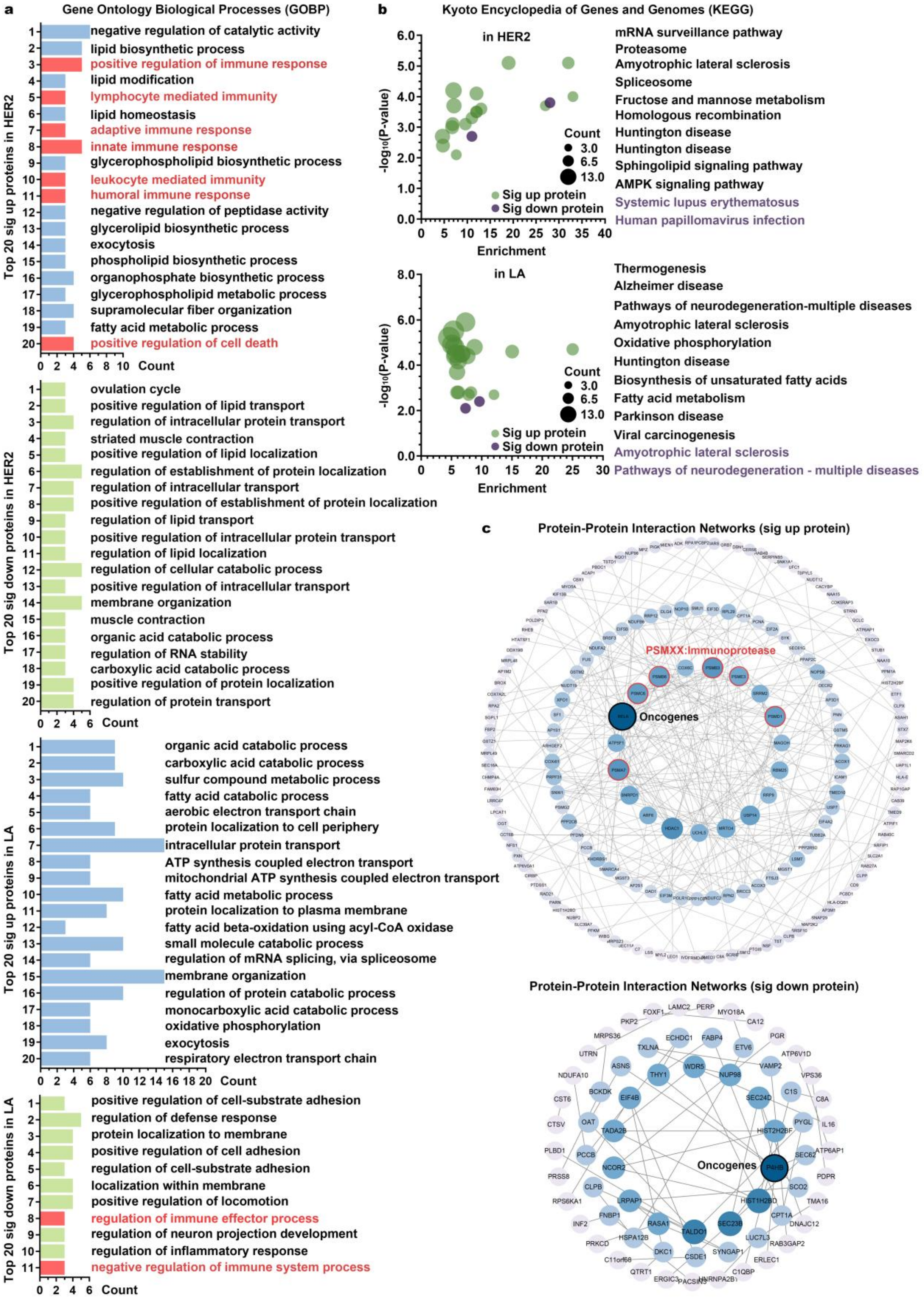

Supplementary Fig. 10 Functional protein signaling pathways and protein-protein interaction network from label-free proteomics data. a, Gene Ontology Biological Processes (GOBP) analysis of proteins with significant differences in expression. Those marked in red were immune-related proteins. b, Kyoto Encyclopedia of Genes and Genomes (KEGG) analysis of significantly different proteins. Those marked in purple were immune-related proteins. c, Protein-Protein Interaction Network (PPI) of significantly different proteins. Proteins with the name PSMXX were immune-related proteins. The Supplementary Fig. 9 and Supplementary Fig.10 illustrated that the proteins with significantly high expression in the bacterial-enrichment region were associated with immunity.

Supplementary Fig. 11

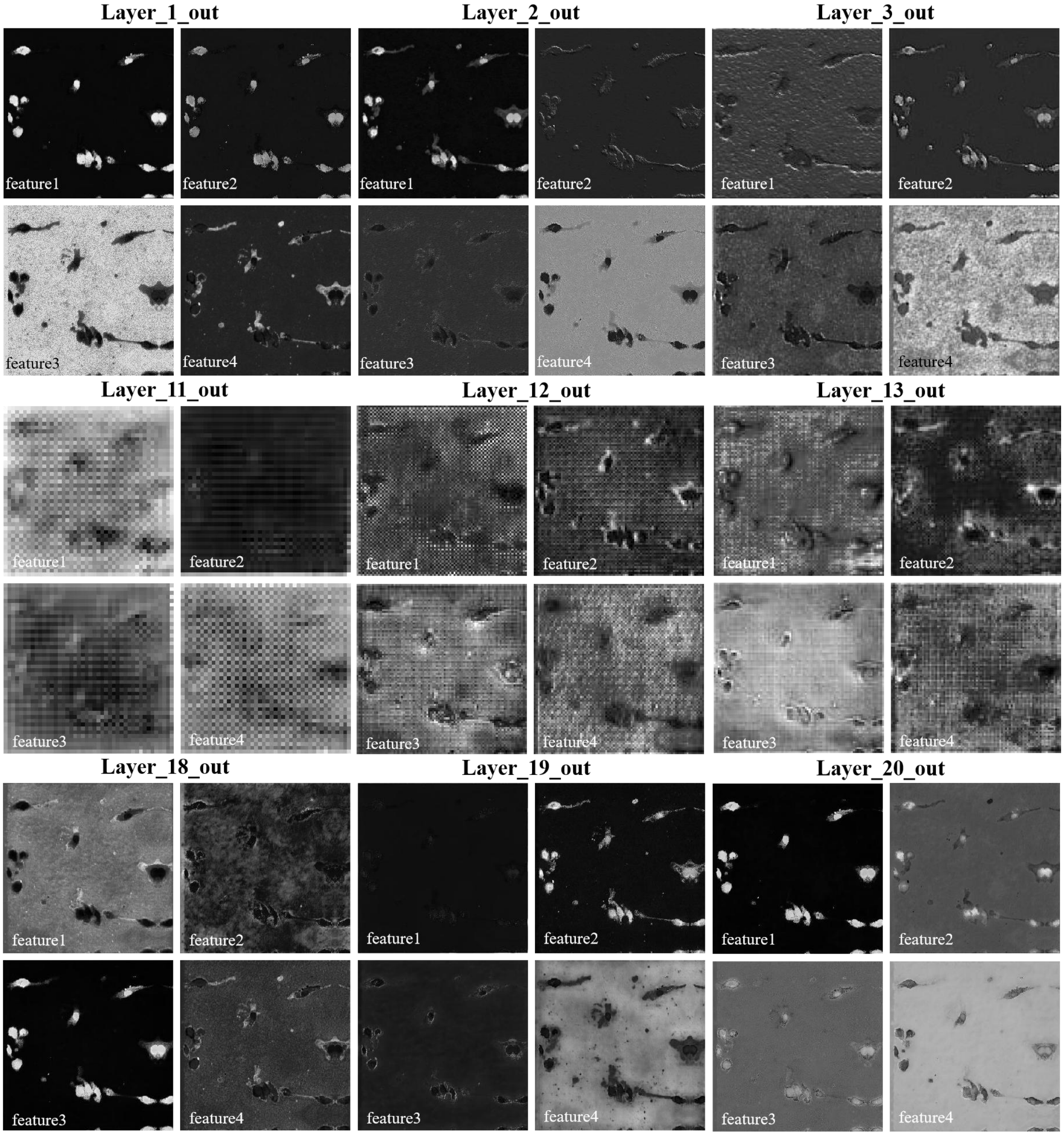

Supplementary Fig. 11 Four features output from each of the 9 middle layers of denoising module. The denoising module had 20 layers and 48 features were incorporated into the training. Four features output from the each of 9 middle layers were shown in Supplementary Fig. 11. It can be seen that the cellular features were effectively captured and maintained as the convolutional network deepens.

Supplementary Fig. 12

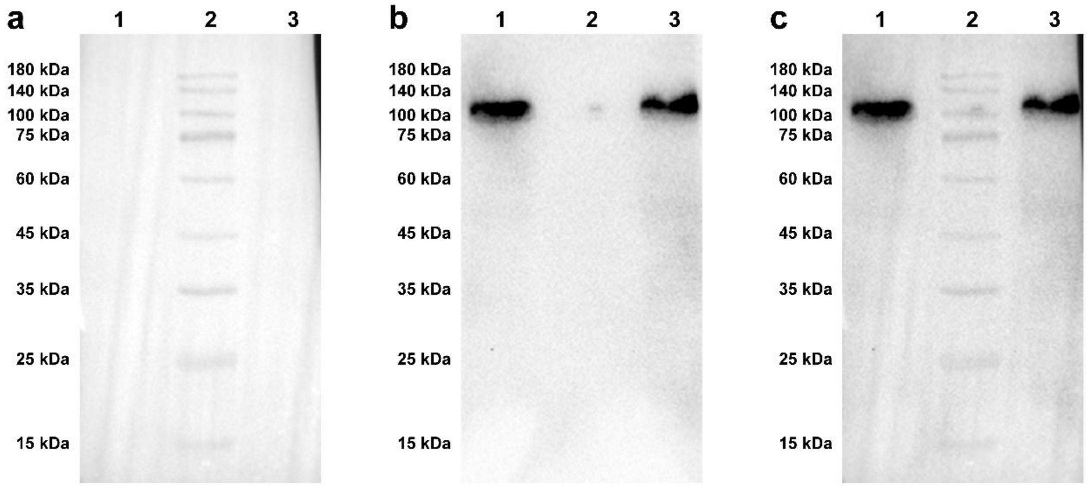

Supplementary Fig. 12 Western blot information. a, Brightfiled image for protein ladder (15~180 kDa). b, Electrochemiluminescence (ECL) signal of CD45. c, merged results of brightfiled image and ECL image. Line 1# and Line 3#: CD45 in two replicates. Line 2# 15~180kDa protein ladder. Source data are provided as a Source Data file.

Supplementary References

1. Kim J, Phan TX, Nguyen VH, Dinh-Vu H, Zheng JH, Yun M, *et al*. Salmonella typhimurium suppresses tumor growth via the proinflammatory cytokine interleukin-1β. Theranostics. 2015;5:1328–42.
2. Lou, X.; Zhang, G.; Herrera, I.;*et al*. Polymer-based elemental tags for sensitive bioassays. Angew. Chem., Int. Ed.46 (32), 6111−6114 (2007).
3. Gallon L, Traitanon O, Yu Y, *et al*. Differential effects of calcineurin and mammalian target of rapamycin inhibitors on alloreactive Th1, Th17, and regulatory T cells. Transplantation. 2015;99(9):1774-1784. https://doi.org/10.1097/TP. 0000000000000717
4. Lyu, Fengye, Han, *et al*. 2023. OmicStudio: A composable Bioinformatics Cloud Platform With Real-Time Feedback That Can Generate High-Quality Graphs for Publication. iMeta. https://doi.org/10.1002/imt2.85
5. Zhou *et al*. Metascape provides a biologist-oriented resource for the analysis of systems-level datasets. Nature Commun. 2019 10(1):1523
6. Jaakko L., Jacob M., Jon H., *et al*. Noise2noise: Learning image restoration without clean data. *In International Conference on Machine Learning*, pages 2965 – 2974, 2018. 1, 2, 3, 5, 6, 7
7. Magauiya Z., Shakarim S., and Se Y.C. Extending stein's unbiased risk estimator to train deep denoisers with correlated pairs of noisy images. *In Advances in Neural Inform*ation Processing Systems, pages 1465 – 1475, 2019. 2, 3, 11
8. Huang, T. , Li, S., Jia, X., *et al*. Neighbor2Neighbor: Self-Supervised Denoising from Single Noisy Images. 2021 IEEE/CVF Conference on Computer Vision and Pattern Recognition (CVPR), Nashville, TN, USA, pp. 14776-14785 (2021).
9. Eom, M., Han, S., Park, P. *et al*. Statistically unbiased prediction enables accurate denoising of voltage imaging data. Nat Methods 20, 1581–1592 (2023).
10. Li, X., Zhang, G., Wu, J. *et al*. Reinforcing neuron extraction and spike inference in calcium imaging using deep self-supervised denoising. Nat Methods 18, 1395 – 1400 (2021).
11. Stringer, C., Wang, T., Michaelos, M. *et al*. Cellpose: a generalist algorithm for cellular segmentation. Nat Methods 18, 100–106 (2021).
