# Supplementary material for: SpiDe-Sr: blind super-resolution network for precise cell segmentation and clustering in spatial proteomics imaging
Source: Nat Commun. 2024 Mar 28;15:2708. doi: 10.1038/s41467-024-46989-z (PMC10978886; doi:10.1038/s41467-024-46989-z)
Supplement: Supplementary file 2 — Reporting Summary [file 41467_2024_46989_MOESM2_ESM.pdf]

nature portfolio

Corresponding author(s): Xianting Ding

Last updated by author(s): Feb 20, 2024

Reporting Summary

Nature Portfolio wishes to improve the reproducibility of the work that we publish. This form provides structure for consistency and transparency in reporting. For further information on Nature Portfolio policies, see our Editorial Policies and the Editorial Policy Checklist.

Statistics

For all statistical analyses, confirm that the following items are present in the figure legend, table legend, main text, or Methods section.

n/a	Confirmed	
☐	☒	The exact sample size (*n*) for each experimental group/condition, given as a discrete number and unit of measurement
☐	☒	A statement on whether measurements were taken from distinct samples or whether the same sample was measured repeatedly
☐	☒	The statistical test(s) used AND whether they are one- or two-sided<br>*Only common tests should be described solely by name; describe more complex techniques in the Methods section.*
☐	☒	A description of all covariates tested
☐	☒	A description of any assumptions or corrections, such as tests of normality and adjustment for multiple comparisons
☐	☒	A full description of the statistical parameters including central tendency (e.g. means) or other basic estimates (e.g. regression coefficient) AND variation (e.g. standard deviation) or associated estimates of uncertainty (e.g. confidence intervals)
☐	☒	For null hypothesis testing, the test statistic (e.g. *F*, *t*, *r*) with confidence intervals, effect sizes, degrees of freedom and *P* value noted<br>*Give P values as exact values whenever suitable.*
☒	☐	For Bayesian analysis, information on the choice of priors and Markov chain Monte Carlo settings
☒	☐	For hierarchical and complex designs, identification of the appropriate level for tests and full reporting of outcomes
☐	☒	Estimates of effect sizes (e.g. Cohen's *d*, Pearson's *r*), indicating how they were calculated

Our web collection on statistics for biologists contains articles on many of the points above.

Software and code

Policy information about availability of computer code

Data collection	Confocal images were acquired on a confocal microscope (LSM 800, Zeiss, German) and saved as 16-bit TIFF images in the ZEN blue 3.3 (Zeiss, German). The images shown in Fig. 2-4 and Supplementary Fig. 3-6 were acquired with 20× /0.40 NA LD PlnN objective. The samples used in Fig. 6 were imaged using 10× /0.3 NA EC PlnN objective and 40× /0.6 NA LD PlnN objective. All IMC images were acquired using a Hyperion laser scanning module coupled to Helios mass cytometer (Fluidigm Sciences). A metal-coated tuning slide (Fluidigm Sciences) was used for optimization of peak intensity and resolution as a function of helium and argon flow. To minimize batch-to-batch variance, a standard internal metal isotope bead was acquired with samples together as a normalization guideline. The acquired raw data was displayed and initially analyzed in MCD Viewer (Fluidigm Sciences) and then saved as 16-bit TIFF images. Then Confocal images were paired with IMC images of the same sample using MATLAB (MATLAB 2019b) scripts.
Data analysis	The confocal images were displayed in the software (ZEN blue, Zeiss, German) and saved as 16-bit tiff images. The acquired raw IMC data was displayed and initially analyzed in MCD Viewer (Fluidigm Sciences) and then saved as 16-bit TIFF images. Then confocal images were paired with IMC images of the same sample using MATLAB (MATLAB 2019b) scripts. https://github.com/DingLabSJTUChenRui/SpiDe-Sr.<br>Data statistics were done in the IBM SPSS Statistics 25.<br>Data visualization was done in the GraphPad Prism 9 or OmicStudio tools.<br>The specific processing was as follows:<br>Step 1 The raw data were imported and displayed in the software (MCD Viewer, Fluidigm), and the valid marker channel of the raw data was selected by an experienced researcher and then stored as 16-bit TIFF format.<br>Step 2 A customized MATLAB script was utilized to collate all images so that the content on each image was an overlay of the nucleus channel and one marker channel. There were 14 markers in each ROI, and 14 images were saved out. The nucleus served primarily for localization.<br>Step 3 All images after collation were super-resolved with SpiDe-Sr.

Step 4 The regions of individual cells in all images were segmented at the pixel level using cytoplasm pattern with adaptive calibration diameter in Cellpose to generate masks. Other default parameters were in Supplementary Table 10. The mask for single-cell segmentation in each ROI was manually adjusted and selected. Single-cell segmentation mask and TIFF images of the 14 channels were overlaid to extract the average expression of markers and spatial features (cell area, perimeter, long-axis length and short-axis length) of single cell using the MATLAB toolbox regionprops. Single-cell marker expressions were summarized by mean pixel values for each channel. The single-cell data were censored at the 99-th percentile to remove outliers, and normalized to the 99-th percentile, as was suggested for these algorithms.
Step 5 Single cells from clinical cohorts were clustered into groups with functionally similar using two unsupervised clustering methods, FlowSOM and PhenoGraph. Both methods were implemented using the python package in the download path provided in the literature. The FlowSOM was repeated 10 times using default parameters within each determined cluster number interval. Every 5 clusters were set as one interval, for a total of 12 intervals between 1 and 60 of cluster numbers. The PhenoGraph was used for the case where the clustering number was not determined, and was repeated 120 times with the nearest neighbour parameter of 30.
Step 6 The clustering results with the highest CH in Step 5 were used for the subsequent analysis. Functionally similar clusters were aggregated into larger groups based on the expression and correlation of markers. For visualization, high-dimensional single-cell data were reduced to two dimensions using the nonlinear dimensionality reduction algorithm t-SNE.
Step 7 Statistical analysis (correlation, difference) was performed on clusters with high expression of G- and G+ bacteria.

For manuscripts utilizing custom algorithms or software that are central to the research but not yet described in published literature, software must be made available to editors and reviewers. We strongly encourage code deposition in a community repository (e.g. GitHub). See the Nature Portfolio guidelines for submitting code & software for further information.

Data

Policy information about availability of data

All manuscripts must include a data availability statement. This statement should provide the following information, where applicable:

- Accession codes, unique identifiers, or web links for publicly available datasets
- A description of any restrictions on data availability
- For clinical datasets or third party data, please ensure that the statement adheres to our policy

https://github.com/DingLabSJTUChenRui/SpiDe-Sr. (DOI:10.5281/zenodo.10669093).

Research involving human participants, their data, or biological material

Policy information about studies with human participants or human data. See also policy information about sex, gender (identity/presentation), and sexual orientation and race, ethnicity and racism.

Reporting on sex and gender	Sex and gender were not used in any scenario as criteria for sample collection.
Reporting on race, ethnicity, or other socially relevant groupings	No race, ethnicity or socially relevant groupings were performed in this study.
Population characteristics	A cohort of 20 patients covering 4 major breast cancer subtypes (HER2, LA, LB, TNBC) .
Recruitment	De-identified tissue samples were collecte with previous patient consent in strict observance of the legal and institutionathical regulations. This was performed by the clinic and no recruitment criteria were used. Because we have no demographic information about either our samples or the patient population, we cannot comment on how any bias may or may not be present. While this would be scientifically helpful, this wall is essential for the patient confidentiality of sensitive procedures and patient privacy trumps our interest.
Ethics oversight	All of our experiments on mouse were ethically proved by Institutional Animal Care and Use Committee (IACUC) of Shanghai Jiao Tong University (approval # 202201309) and the experiments on human samples were ethically cleared by Institutional Review Board for Human Research Protections of Shanghai Jiao Tong University (approval # B2022357P).

Note that full information on the approval of the study protocol must also be provided in the manuscript.

Field-specific reporting

Please select the one below that is the best fit for your research. If you are not sure, read the appropriate sections before making your selection.

[x] Life sciences [ ] Behavioural & social sciences [ ] Ecological, evolutionary & environmental sciences

For a reference copy of the document with all sections, see nature.com/documents/nr-reporting-summary-flat.pdf

Life sciences study design

All studies must disclose on these points even when the disclosure is negative.

Sample size	Samples were collected until the sample size was sufficient to give comparison and reliable estimates. No sample size calculations were performed. No statistical method was used to predetermine sample size.
Data exclusions	No data were artificially excluded from the analysis.

Replication	As the laser ablation during IMC imaging process precludes the same sample being acquired twice, IMC experiments were completed once for all samples. For consistency, confocal experiments were also snapped once for all samples. However, for each sample, we generated several biological replicates when it was possible. For MCF-7 cell, we analyzed n = 814 paired cells; For mouse fatty tissue, we analyzed n = 860 paired cells; For breast cancer tissue, we analyzed n = 691 paired cells.
Randomization	No randomization was performed, as this was not a case-control study.
Blinding	As there was no therapeutic intervention in this study, no blinding was performed.

Reporting for specific materials, systems and methods

We require information from authors about some types of materials, experimental systems and methods used in many studies. Here, indicate whether each material, system or method listed is relevant to your study. If you are not sure if a list item applies to your research, read the appropriate section before selecting a response.

Materials & experimental systems

n/a	Involved in the study
☐	☒ Antibodies
☐	☒ Eukaryotic cell lines
☒	☐ Palaeontology and archaeology
☐	☒ Animals and other organisms
☒	☐ Clinical data
☒	☐ Dual use research of concern
☒	☐ Plants

Methods

n/a	Involved in the study
☒	☐ ChIP-seq
☒	☐ Flow cytometry
☒	☐ MRI-based neuroimaging

Antibodies

Antibodies used

Antibody information is supplied in Supplementary Table1 1, which contains information about antigen; clone; vender; catalog number and working concentration as follows:
Tubulin; /; Beyotime; AF1216; 20 µg/mL;
CD45; /; Beyotime; AF7839; 20 µg/mL;
CD34; /; Beyotime; AF1387; 20 µg/mL;
Donkey anti-rabbit secondary antibody; Poly4064; Biolegend; 406416; 10 µg/mL;
PKCD; EPR17075; Abcam; ab222229, 5 µg/mL;
PR; YR85; Abcam; ab206926), 1 µg/mL;
IFI6; /; Abcam; ab192314; 5 µg/mL;
HER2; EP1045Y; Abcam; ab194979, 0.25 µg/mL;
Ki67; SP6; Abcam; ab197547; 0.15 µg/mL;
ISG15; /; Abcam; ab285370; 5 µg/mL;
ER; EPR4097; Abcam; ab167610, 3 µg/mL;
ZC3HAV1; /; Abcam; ab154680; 5 µg/mL;
LPS; WN1 222-5; HycultBiotech; HM6011; 1 µg/mL;
LTA; mAb 55; HycultBiotech; HM2048; 0.5 µg/mL;
CD45; HI30; Biolegend; 304045; 0.15 µg/mL;
CD68; Abcam; 283667; 5 µg/mL;
CD8a; CAL66; Abcam; ab251596; 1 µg/mL;
CD19; 6OMP31; Invitrogen; 14-0194-95; 2.5 µg/mL.

Validation

Primary antibodies were validated by both the manufacturers (showed below) and negative control experiments omitting the primary antibody.
Antigen; species; applications; validation from company's website; relevant citations
β-Tubulin; Rabbit; WB, IF, IHC, ICC, FC; https://www.beyotime.com/product/AF1216.htm;
CD45; Rabbit; WB, IF, IHC; https://www.beyotime.com/product/AF7839.htm;
CD34; Rabbit; WB, IP, IF, IHC, ICC; https://www.beyotime.com/product/AF1387.htm;
PKCD; Rabbit; WB, IHC-P, ICC/IF, Flow Cyt (Intra); https://www.abcam.cn/products/primary-antibodies/pkc-delta-antibody-epr17075-bsa-and-azide-free-ab222229.html; https://onlinelibrary.wiley.com/doi/10.1002/cyto.a.24550;
PR; Rabbit; IHC-P, IP, WB, Flow Cyt (Intra), ICC/IF; https://www.abcam.cn/products/primary-antibodies/progesterone-receptor-antibody-yr85-bsa-and-azide-free-ab206926.html; https://onlinelibrary.wiley.com/doi/10.1002/cyto.a.24550;
IFI6; Rabbit; WB, IHC-P, ICC/IF; https://www.abcam.cn/products/primary-antibodies/ifi6-antibody-ab192314.html; https://onlinelibrary.wiley.com/doi/10.1002/cyto.a.24550;
HER2; Rabbit; WB, ICC/IF, IHC-P, IP; https://www.abcam.cn/products/primary-antibodies/erbb2--her2-antibody-ep1045y-bsa-and-azide-free-ab194979.html; https://onlinelibrary.wiley.com/doi/10.1002/cyto.a.24550;
Ki67; Rabbit; WB, mIHC, Flow Cyt (Intra), IHC-P, ICC/IF; https://www.abcam.cn/products/primary-antibodies/ki67-antibody-sp6-bsa-and-azide-free-ab197547.html; https://onlinelibrary.wiley.com/doi/10.1002/cyto.a.24550;

ISG15; Rabbit; WB, Flow Cyt (Intra), IHC-P, ICC/IF, IP; https://www.abcam.cn/products/primary-antibodies/isg15-antibody-epr24482-49-bsa-and-azide-free-ab285370.html; https://onlinelibrary.wiley.com/doi/10.1002/cyto.a.24550;
ER; Rabbit; Flow Cyt (Intra), ChIC/CUT&RUN-seq, IHC-P, WB, IHC-Fr, ICC/IF; https://www.abcam.cn/products/primary-antibodies/estrogen-receptor-alpha-antibody-epr4097-bsa-and-azide-free-ab167610.html; https://onlinelibrary.wiley.com/doi/10.1002/cyto.a.24550;
ZC3HAV1, Rabbit; WB, IHC-P, ICC/IF; https://www.abcam.cn/products/primary-antibodies/zinc-finger-antiviral-protein-antibody-ab154680.html?productWallTab=ShowAll; https://onlinelibrary.wiley.com/doi/10.1002/cyto.a.24550;
LPS; Mouse; WB, Elisa, IHC-P; https://www.hycultbiotech.com/product/lipopolysaccharide-core-mab-wn1-222-5/; https://onlinelibrary.wiley.com/doi/10.1002/cyto.a.24550;
LTAL Mouse; Flow cytometry, Frozen sections, Immuno assays, Immuno fluorescence, Western blot; https://www.hycultbiotech.com/product/hm2048-200ug/; https://onlinelibrary.wiley.com/doi/10.1002/cyto.a.24550;
CD45; Mouse; FC, CyTof (additional reported applications include IHC-P); https://www.biolegend.com/en-us/products/purified-anti-human-cd45-maxpar-ready-antibody-9188; https://onlinelibrary.wiley.com/doi/10.1002/cyto.a.24550;
CD68; Rabbit; IHC-P, ICC/IF, WB, IHC-Fr, Flow Cyt (Intra); https://www.abcam.cn/products/primary-antibodies/cd68-antibody-epr23917-164-bsa-and-azide-free-ab283667.html; https://onlinelibrary.wiley.com/doi/10.1002/cyto.a.24550;
CD8a; Rabbit; ICC/IF, IP, Flow Cyt, IHC-P, mIHC; https://www.abcam.cn/products/primary-antibodies/cd8-alpha-antibody-cal66-bsa-and-azide-free-ab251596.html; https://onlinelibrary.wiley.com/doi/10.1002/cyto.a.24550;
CD19 ; Rat; WB, IHC, IHC(P), IHC(F), ICC/IF, Flow; https://www.thermofisher.cn/cn/zh/antibody/product/CD19-Antibody-clone-6OMP31-Monoclonal/14-0194-95; https://onlinelibrary.wiley.com/doi/10.1002/cyto.a.24550.

Donkey anti-rabbit secondary antibody (Biolegend, 406416) for fluorescent/metal dual-labeled immunostaining has been validated by the manufacturer to be suitable for this publication.

Eukaryotic cell lines

Policy information about cell lines and Sex and Gender in Research

Cell line source(s)	MCF-7 cells were obtained from ATCC (HTB-22).
Authentication	The MCF-7 cell line was sent to a biotechnology company for STR (Short Tandem Repeat) cell line identification. The identification report showed that our cell line was indeed MCF-7 cell line. The STR identification report was uploaded as an additional supplementary file.
Mycoplasma contamination	Cell lines were not tested for mycoplasma contamination.
Commonly misidentified lines (See ICLAC register)	CVCL_1452.

Animals and other research organisms

Policy information about studies involving animals; ARRIVE guidelines recommended for reporting animal research, and Sex and Gender in Research

Laboratory animals	Wildtype C57BL/6J mice around 6 weeks were used in this study. Mice were housed in a pathogen-free colony with 22-24°C temperature and in 40-70% relative humidity environment. The mice were given access to food and water ad libitum with a 12hr light/dark cycle.
Wild animals	No wild animals were used in this study.
Reporting on sex	Sex was not considered in our study design and both male and female samples were used.
Field-collected samples	No field-collected samples were used in this study.
Ethics oversight	All of our experiments on mouse were ethically proved by Institutional Animal Care and Use Committee (IACUC) of Shanghai Jiao Tong University (approval # 202201309)

Note that full information on the approval of the study protocol must also be provided in the manuscript.

Plants

Seed stocks	*Report on the source of all seed stocks or other plant material used. If applicable, state the seed stock centre and catalogue number. If plant specimens were collected from the field, describe the collection location, date and sampling procedures.*
Novel plant genotypes	*Describe the methods by which all novel plant genotypes were produced. This includes those generated by transgenic approaches, gene editing, chemical/radiation-based mutagenesis and hybridization. For transgenic lines, describe the transformation method, the number of independent lines analyzed and the generation upon which experiments were performed. For gene-edited lines, describe the editor used, the endogenous sequence targeted for editing, the targeting guide RNA sequence (if applicable) and how the editor was applied.*
Authentication	*Describe any authentication procedures for each seed stock used or novel genotype generated. Describe any experiments used to assess the effect of a mutation and, where applicable, how potential secondary effects (e.g. second site T-DNA insertions, mosiacism, off-target gene editing) were examined.*
